# Thermoelectric properties of a quantum dot attached to normal metal and topological superconductor

**DOI:** 10.1038/s41598-024-84770-w

**Published:** 2025-01-24

**Authors:** Piotr Trocha, Thibaut Jonckheere, Jérôme Rech, Thierry Martin

**Affiliations:** 1 Institute of Spintronics and Quantum Information, Faculty of Physics and Astronomy, Adam Mickiewicz University, 61-614 Poznań, Poland; 2 Aix Marseille Univ, Université de Toulon, CNRS, CPT, Marseille, France

**Keywords:** Energy science and technology, Nanoscience and technology, Physics, Condensed-matter physics, Superconducting properties and materials, Topological matter

## Abstract

The thermoelectric properties of hybrid systems based on a single-level quantum dot coupled to a normal-metal/half-metallic lead and attached to a topological superconductor wire are investigated. The topological superconductor wire is modeled by a spinless p-wave superconductor which hosts both a Majorana bound state at its extremity and above gap quasiparticle excitations. The main interest of our investigation is to study the interplay of sub-gap and single-particle tunneling processes and their contributions to the thermoelectric response of the considered system. The above gap tunneling driven by a temperature gradient is responsible for relatively large thermopower, whereas sub-gap processes only indirectly influence the thermoelectric response. The thermoelectric coefficients, including electric conductance, Seebeck coefficient (thermopower), heat conductance, and figure of merit, are calculated by means of the non-equilibrium Green’s function technique and the temperature dependence of the superconducting gap is considered within the BCS theory. We also consider the system out of equilibrium working as a heat engine. The output power and the corresponding efficiency are presented. Interestingly, under certain conditions, it is possible to extract more power in the superconducting phase than in the normal phase, with comparable efficiency.

## Introduction

Thermoelectricity has attracted renewed interest since the discovery of the spin Seebeck effect in metallic magnets^1^. Especially, great expectations are placed into thermoelectric phenomena of nanoscale systems as they may reveal efficient ways of converting heat into electric energy, inaccessible in conventional materials obeying Wiedemann-Franz law^2^ and Mott relation^3^.

The above problems can be overcome by using nanoscale systems in which due to their peculiar properties arising from space quantization (level quantization), an enhancement of the thermoelectric response is possible ^4–11^. Generally, there are two main factors of space quantization leading to the enlargement of the figure of merit in nanoscale systems. First, such systems (due to space quantization) may exhibit violation of the Wiedemann-Franz law^12,13^. Secondly, they usually show a reduced thermal conductance^14^. These discoveries initiated both experimental^15–19^ and theoretical^20–31^ investigations on thermoelectric properties of nanoscale systems including quantum dots (QDs).

Quantum dots (QDs) can be viewed as promising low-dimensional system revealing a discrete structure of the density of states, and thus, possessing the above properties^32^. Moreover, as the parameters of QDs can be easily tunable, the thermoelectric devices based on QDs seem to be even more promising candidates for energy conversion devices. Moreover, multi-dot structures reveal a variety of different interference effects, including Fano and Dicke phenomena^33–35^, which further amplify the thermoelectric efficiency^36^. Apart from that, Coulomb correlations in QD can also influence its thermoelectric effects in the Kondo regime^37–44^.

Incorporating external magnetic leads into the thermoelectric system may result in spin-dependent Seebeck effect related to voltage generation by a temperature gradient. Such an effect has been observed for the first time in thin film metallic magnets^1,45^ and further in tunnel junction^46,47^. Spin-dependent thermoelectric phenomena have also been investigated theoretically in QD-based systems attached to ferromagnetic leads ^21,22,36,48^.

As superconductors perfectly conduct electric current and simultaneously, are poor thermal conductors, it seems that they should be excellent thermoelectric materials. However, they show a very weak thermoelectric response at low temperature which is attributed to particle-hole symmetry^49,50^. It turned out, that in hybrid systems^51–55^, consisting of QDs coupled to a normal metal and a superconductor, this problem can be somehow overcome and a relatively large thermoelectric response can be expected^56^. However, most investigations have been devoted to normal metal-superconductor and superconductor-superconductor junctions working as heat engines^57–62^, refrigerators^63–74^, heat transistors^75^ and diodes^76^. Moreover, boosted thermoelectric conversion has been proposed in a three-terminal device with normal and superconducting leads^77^ and observed in superconductor-ferromagnet tunnel junctions^78,79^.

Apart from this, thermoelectric properties of QD hybrid systems with *s*-wave superconductor were also explored, both regarding finite gap models^80–85^ and in the Andreev transport regime, where thermoelectricity occurs between normal-metal leads in the presence of an additional superconducting contact considered in the large gap approximation^86–89^. Moreover, it has been shown that Cooper beam splitters based on QD systems can work as a heat engine or a refrigerator with efficiency close to the Carnot limit^90,91^.

The possible existence of Majorana bound states (MBS) in topological superconductor nanowire^92–94^ has initiated broad investigations on transport properties of such systems. Theoreticians have been looking for signatures of MBS in the transport characteristics of devices such as electronic current and noise, containing one or several superconductors^95–98^. However, in the case of hybrid structures containing a topological superconductor most early attention has been paid to the low energy limit in which only the MBS sector survives^99,100^. It turns out that such a hybrid system, with one normal metal lead coupled to a topological superconductor through some nanoscopic structure, e. g. a quantum dot, does not exhibit any thermoelectric response due to particle-hole symmetry. More specifically, it has been shown that in a junction consisting of a Majorana nanowire, hosting only MBS states, whose ends are tunnel-coupled to normal contacts, there is no Seebeck effect^101^. This results from the aforementioned particle-hole symmetry. Therefore, most theoretical studies have focused on the influence of MBS on the thermoelectric response of the hybrid quasi-three-terminal topological systems in which thermoelectricity occurs between normal metal leads^102–118^. In these papers the topological nanowire has been described by effective low energy models considering only the MBS. For this reason, here we propose a more realistic model for the topological superconductor based on Ref.^95^ which includes not only the MBS but also the quasiparticle states above the superconducting gap. Including these above gap quasiparticle states allows for a finite thermoelectric response in a two-terminal system consisting of a normal metal coupled to a topological superconductor, which is not possible considering only MBSs. Nevertheless, sub-gap states may still indirectly influence the thermoelectric response (as will be shown later). The thermoelectric properties of QD systems coupled to topological nanowires described by finite gap models seems to be unexplored^119^. Therefore, here we fill this drawback by considering a finite gap model. To induce thermoelectricity particle-hole symmetry has to be broken. This can be simply realized by tuning the dot’s energy level by applying proper gate voltage. Thus, quasiparticle states are essential for generating thermopower. Note that the subgap states remain symmetric with respect to zero energy, even when the dot energy level is shifted away from zero. As a result, subgap states cannot generate thermoelectricity and above the gap states must be considered. Here, we must also point out that several proposals suggest that electron-hole symmetry can be broken if the superconductor is brought into proximity with ferromagnetic contacts^120^ or by combining an external magnetic field with a spin filter^121^. This particle-hole symmetry breaking arises from an exchange field-induced splitting of the spin-up and spin-down energy subbands in the superconductor. Consequently, large thermoelectric effects are predicted. Moreover, odd-frequency superconductivity allows for the observation of a finite thermoelectric response in the subgap regime^122^. The breaking of particle-hole symmetry is achieved by applying a magnetic field in conjunction with a finite spin polarization of the ferromagnetic lead attached to the quantum dot. These results suggest that breaking particle-hole symmetry would beneficially affect the thermoelectric response of the system in the subgap regime. However, in the present system breaking particle-hole symmetry would destroy MBS and the spinfull model would be required to introduce this effect, which is beyond the scope of the present paper.

Moreover, we show that a hybrid structure based on normal metal/QD/topological nanowire allows us to obtain a remarkable thermoelectric response not possible with a conventional superconductor^49,50^. Apart from that, we indicate the differences in thermoelectric response of the considered system with respect to the one with an *s-wave* electrode instead of a topological superconductor. Noticeably, a topological Josephson heat engine has been proposed in Ref.^123^ which also suggests that the topic warrants investigation.

The considered system can be realized using a semiconductor nanowire with strong spin-orbit coupling (e. g. InSb or InAs nanowires) brought into proximity with an s-wave superconductor (e. g. niobium titanium nitride (NbTiN) or aluminum) and connected to a normal metal electrode (e. g. gold). By applying gate voltages to the electrodes, a quantum dot can be formed in the end segment of the nanowire that is in contact with the normal metal^94^. Then, by applying a magnetic field *B* parallel to the nanowire and satisfying the condition $g \mu _B B/2 > \sqrt{\mu ^2 + \Delta ^2}$, Majorana states can emerge at the ends of the nanowire. Here, $\Delta$ denotes the superconducting gap, *g* is the Landé g-factor (for bulk InSb, $g \approx 50$^93^), and $\mu$ is the chemical potential of the nanowire.

The paper is organized in the following way. In Theoretical background we present the theoretical description of the considered system. Particularly, we introduce the model taken into consideration and derive the formulas for the thermoelectric coefficients in the linear response regime. The numerical results are presented and discussed in Results. This section is divided into three parts. In the first part we describe the transmission coefficients of the system, whereas in the second part, we present the thermoelectric coefficients in the linear response regime. In the last part, results on the non-equilibrium phenomena emerging from a temperature bias are presented. Finally, the paper is concluded in Conclusions.

## Theoretical background

### Theoretical background

**Figure 1:**
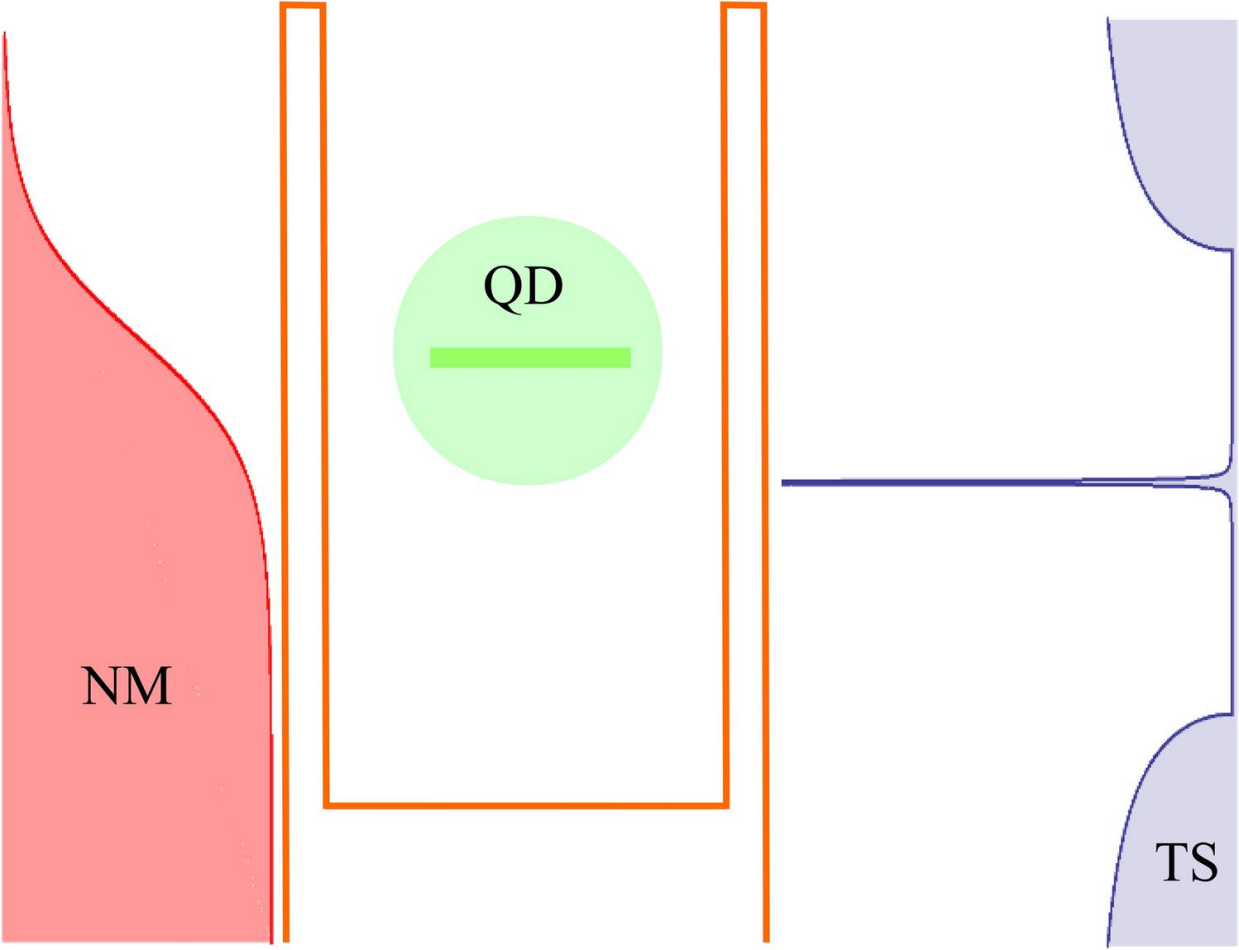
Schematic description of the setup. A Fermi function (red) on the left hand side represents the normal metal, while the density of states of the topological superconductor is depicted on the right hand side (purple). The density of states of a topological nanowire consists of a narrow peak at zero energy, attributed to the Majorana bound state, as well as quasiparticle energy bands for $|\varepsilon | \ge \Delta$, where $\Delta$ represents the superconducting gap. The central region (green) represents a single level quantum dot, which is coupled to the two leads by tunnel couplings depicted by the two barriers surrounding the dot.

We consider a system consisting of a quantum dot (QD) coupled to two external leads. One of the electrodes is a normal metal whereas the second consists of a topological superconductor (TS) wire. The TS wire is modeled as a spinless p-wave superconductor which hosts both Majorana bound states and continuum quasiparticle excitations. The setup is described in Fig. 1, where the normal metal corresponds to the temperature smoothed Fermi function on the left, the single level dot is the central region, and the topological superconductor density of states is on the right.

The whole system can be modeled by the Hamiltonian of the following form,

1 $$\begin{aligned} H=H_{c}+H_{QD}+H_{T_N}+H_{T_{TS}}. \end{aligned}$$

The first term, $H_{c}$, represents the Hamiltonian of the external leads: $H_{c}=H_L +H_R$. Here, $H_L=\sum _{{\textbf{k}}}\varepsilon _{{\textbf{k}}L} c^\dag _{{\textbf{k}}L}c_{{\textbf{k}}L}$ describes the normal metal lead in the non-interacting quasiparticle approximation, assumed to be on the left (L) side, with $\varepsilon _{{\textbf{k}}L}$ representing the electronic spectrum. In turn, $H_R$ stands for the Hamiltonian of the TS (right) lead described by a spinless single-channel p-wave superconductor, corresponding to the low-energy limit of a Kitaev chain,

2 $$\begin{aligned} H_R=\int _{0}^{\infty }dx\Psi _{TS}^\dag (x)(-iv_F\partial _x\sigma _z+\Delta \sigma _y)\Psi _{TS}(x), \end{aligned}$$

where $\sigma _j (j=x,y,z)$ are Pauli matrices in Nambu space. Here, we choose the proximity-induced gap $\Delta$ as a real positive number and $\Psi _{TS}=(c_r,c_l^\dag )^T$ is the Nambu spinor with right- and left-moving fermion operators. The boundary Green’s function of the TS for a semi-infinite Kitaev chain, reads

3 $$\begin{aligned} \textbf{g}_{TS}(t-t')=-i\langle \mathcal {T_C}\Psi ^\dag (t)\Psi (t') \rangle , \end{aligned}$$

with the Nambu spinor $\Psi =(c,c^\dag )^T$ for $c=[c_l+c_r](x\rightarrow 0)$ and $\mathcal {T_C}$ stands for Keldysh time ordering. The boundary retarded/advanced Green’s function acquires the following form in the wideband limit^95^,

4 $$\begin{aligned} \textbf{g}_{TS}^{r/a}(\varepsilon )=\pi \rho _N\frac{[\theta (\Delta -|\varepsilon |)\sqrt{\Delta ^2-\varepsilon ^2}\mp i~\textrm{sgn}(\varepsilon )\theta (|\varepsilon |-\Delta )\sqrt{\varepsilon ^2-\Delta ^2}]\sigma _0+\Delta \sigma _x}{\varepsilon \pm i0^+}. \end{aligned}$$

with $\rho _N$ being the density of states in the normal state (assumed independent of energy). The corresponding density of states matrix is,

5 $$\begin{aligned} \check{\rho } (\varepsilon )=\pi \rho _N\left[ [\Delta (\sigma _0+\sigma _x)]\delta (\varepsilon )+\frac{\theta (|\varepsilon |-\Delta )\sqrt{\varepsilon ^2-\Delta ^2})}{\pi |\varepsilon |}\sigma _0\right] , \end{aligned}$$

whereas the density of states $\rho _R(\varepsilon )=[{\check{\rho } (\varepsilon )}]_{11}$ has the form

6 $$\begin{aligned} \rho _R (\varepsilon )=\pi \rho _N\left[ \frac{\theta (|\varepsilon |-\Delta )\sqrt{\varepsilon ^2-\Delta ^2})}{\pi |\varepsilon |}+\Delta \delta (\varepsilon )\right] . \end{aligned}$$

Note that the zero-energy Majorana state is a feature resulting from intrinsic properties of the topological superconductor. In particular, the origin of this state is different from Andreev states occurring due to the proximity effect of the s-wave superconductor and normal metal.

The Green’s function describing the normal lead is given by,

7 $$\begin{aligned} \textbf{g}_{N}^{r/a}(\varepsilon )=\mp i\pi \rho _L\sigma _0. \end{aligned}$$

The non-interacting quantum dot is described by the following Hamiltonian:

8 $$\begin{aligned} H_{QD}=\varepsilon _d d^\dag d, \end{aligned}$$

where $\varepsilon _d$ denotes the energy level of the dot. The corresponding Green’s function has the form, $[\textbf{g}_d^{r/a}(\varepsilon )]^{-1}=\textrm{diag}(\varepsilon -\varepsilon _d\pm i0^+,\varepsilon +\varepsilon _d\pm i0^+)$

The last two terms of the Hamiltonian (1) describe the tunneling of electrons between the leads and the dot. These terms can be written as,

9 $$\begin{aligned} H_{T_N}=\sum _{\textbf{k}} \limits (V_{\textbf{k}}^L c^\dag _{\textbf{k}}d+\mathrm H.c.), \end{aligned}$$

for the coupling to the normal metal lead and as,

10 $$\begin{aligned} H_{T_{TS}}= V^R c^\dag (0) d +\mathrm H.c., \end{aligned}$$

for the coupling to the TS lead, with $V_{\textbf{k}}^i$, for $i=L,R$, denoting the relevant tunneling matrix elements between the dot and *i*-th lead. Furthermore, we assume that these matrix elements are independent of $\textbf{k}$, $V_{\textbf{k}}^i \equiv V^i$. The coupling of the dot to the normal metal lead, $i=L$, can then be parameterized by the tunneling rate $\Gamma _{L}=2\pi |V^{L}|^2\rho _{L}$, where $\rho _{L}$ is the density of states in the lead *L*. Similarly, the coupling to the TS electrode in the corresponding normal state is denoted as $\Gamma _{R}=2\pi |V^{R}|^2\rho _{R}$, and is independent of spin orientation and of energy. This coupling becomes modified in the superconducting state, as will be described in the following.

The coupling matrix elements in Nambu space can be written as

11 $$\begin{aligned} \textbf{V}^{i}=V^i\sigma _{z}. \end{aligned}$$

The corresponding retarded/advanced self-energies of the dot can be found from,

12 $$\begin{aligned} \varvec{\Sigma }_{i}^{r/a}= \textbf{V}^{i\dag }\textbf{g}_{i}^{r/a}\textbf{V}^{i} \end{aligned}$$

where $\textbf{g}_{i}^{r/a}$ is the retarded/advanced Green’s function for the *i*th lead defined above. In turn, the coupling matrix $\varvec{\Gamma }_{\beta }$ (for $\beta =L,R$) is derived from

13 $$\begin{aligned} \varvec{\Gamma }_{\beta }=i[\varvec{\Sigma }^{r}_{\beta }-\varvec{\Sigma }^{a}_{\beta }]. \end{aligned}$$

The retarded Green’s function describing the system $\textbf{G}^{r}_{\sigma }(\varepsilon )$ has been obtained from the Dyson equation

14 $$\begin{aligned} \textbf{G}^{r}_{d}=[(\textbf{g}^r_d)^{-1}-\varvec{\Sigma }^r ]^{-1}, \end{aligned}$$

with $\textbf{g}^r_{d}$ denoting the retarded Green’s function of the dot isolated from the leads, and $\varvec{\Sigma }^r =\varvec{\Sigma }^r_{L}+\varvec{\Sigma }^r_{R}$ representing the retarded self-energy which describes tunneling between the QD and electrodes. The retarded self-energy due to the coupling to the normal metal lead, $\varvec{\Sigma }^r_{L}$, taken in the wide band approximation acquires the form

15 $$\begin{aligned} \varvec{\Sigma }^{r}_{L}= -\frac{i}{2}\Gamma _L\sigma _0 , \end{aligned}$$

whereas the self-energy due to the coupling to the right (TS) lead takes the form

16 $$\begin{aligned} \varvec{\Sigma }^{r}_{R}= \frac{\Gamma _R}{2\pi \rho _N}\sigma _{z}\textbf{g}_{TS}^r(\varepsilon )\sigma _{z}. \end{aligned}$$

The charge current $J_L^e$ flowing in a biased system from left to right can be calculated from the following formula^124^:

17 $$\begin{aligned} J_e=\frac{ie}{2\hbar }\int \frac{\textrm{d}\varepsilon }{2\pi } \{[\varvec{\Gamma }_{L}-\varvec{\Gamma }_{R}] \textbf{G}^{<} (\varepsilon ) +[\varvec{\Gamma }_{L}\textbf{f}_L(\varepsilon )- \varvec{\Gamma }_{R}\textbf{f}_R(\varepsilon )] {[}\textbf{G}^{r}(\varepsilon )-\textbf{G}^{a}(\varepsilon )]\}_{11}, \end{aligned}$$

with $\textbf{f}_{L} (\varepsilon )$ describing the Fermi-Dirac distribution in the normal metal lead,

18 $$\begin{aligned} \textbf{f}_{L} = \left( \begin{array}{cccc} f^L_{\varepsilon -\mu _L} & 0 \\ 0 & f^L_{\varepsilon +\mu _L} \\ \end{array} \right) , \end{aligned}$$

while $\textbf{f}_{R}=f^R_\varepsilon \,\textrm{diag}(1,1)$ is the Fermi distribution for the TS lead. Here, we assumed the TS to be grounded, i. e. $\mu _R=0$, and introduced the notation $f^\mu _\epsilon = 1/\left( 1+e^{\epsilon /T_\mu }\right)$. Note that due to current conservation $J_e\equiv J_{e}^L=-J_{e}^R$.

To derive the lesser Green’s function we apply the Keldysh relation,

19 $$\begin{aligned} \textbf{G}^{<}=\textbf{G}^{r}\varvec{\Sigma }^{<}\textbf{G}^{a}, \end{aligned}$$

whereas the lesser self-energy can be obtained from the following formula:

20 $$\begin{aligned} \varvec{\Sigma }^{<}=\varvec{\Sigma }^{<}_{L}+\varvec{\Sigma }^{<}_{R}= i(\textbf{f}_L\varvec{\Gamma }_{L}+ \textbf{f}_R\varvec{\Gamma }_{R}), \end{aligned}$$

which is valid for non-interacting QDs and also for interactions taken in one-body approximations.

Making use of the above formulas and taking into account the identity, $\textbf{G}^{r}-\textbf{G}^{a}=-i\textbf{G}^{r}\varvec{\Gamma }\textbf{G}^{a}$, with $\varvec{\Gamma }=\varvec{\Gamma }_{L}+\varvec{\Gamma }_{R}$, the current can be written in the form of a Landauer-like formula

21 $$\begin{aligned} J_{e}=\frac{e}{h}\int \textrm{d}\varepsilon \left[ (f_{\varepsilon -\mu _{L}}^{L}-f_{\varepsilon +\mu _{L}}^{L})\mathcal {T}_{LL}^{A}(\varepsilon )\right. +\left. (f_{\varepsilon -\mu _{L}}^{L}-f_{\varepsilon -\mu _{R}}^{R})\mathcal {T}_{LR}^{S}(\varepsilon ) \right] , \end{aligned}$$

with $\mathcal {T}_{LL}^A(\varepsilon )=G_{12}^r[{\varvec{\Gamma }}^L{\textbf{G}}^a{\varvec{\Gamma }}^L]_{21}=\Gamma _{L}^{2}|G_{12}^r|^2$ and $\mathcal {T}_{LR}^S(\varepsilon )=[\textbf{G}^r\varvec{\Gamma }^R\textbf{G}^a\varvec{\Gamma }^L]_{11}$. The first term represents Andreev processes, while the second one describes quasiparticle transfer. It is straightforward to generalize these results in order to obtain the heat current:

22 $$\begin{aligned} J_{Q}= & \frac{1}{h}\int \textrm{d}\varepsilon (\varepsilon -\mu _L) (f_{\varepsilon -\mu _{L}}^{L}-f_{\varepsilon -\mu _{R}}^{R})\mathcal {T}_{LR}^{S}(\varepsilon ) -\frac{\mu _L}{h}\int \textrm{d}\varepsilon (f_{\varepsilon -\mu _{L}}^{L}-f_{\varepsilon +\mu _{L}}^{L})\mathcal {T}_{LL}^{A}(\varepsilon ). \end{aligned}$$

Note that Andreev-like processes transfer heat only for a biased system i. e. for $\mu _L\ne 0$.

### Linear response regime

Assuming the chemical potential and the temperature of the left electrode to be $\mu _L=\mu +\delta \mu$ and $T_L=T+\delta T$ respectively, whereas those of the right electrode are $\mu _R=\mu$ and $T_R=T$, with $\delta \mu$ and $\delta T$ denoting infinitesimally small quantities, the charge current, $J_e$, in the linear response regime becomes

23 $$\begin{aligned} J_{e}= & \frac{e}{h}\int \textrm{d}\varepsilon \left( -\frac{df}{d\varepsilon }\right) \left[ 2\mathcal {T}_{LL}^{A}\delta \mu +\mathcal {T}_{LR}^{S}(\varepsilon )\delta \mu + \left( \frac{\varepsilon -\mu }{T}\right) \mathcal {T}_{LR}^{S}(\varepsilon )\delta T\right] . \end{aligned}$$

Analogously, one can obtain the heat current

24 $$\begin{aligned} J_{Q}= & \frac{1}{h}\int \textrm{d}\varepsilon (\varepsilon -\mu ) \left( -\frac{df}{d\varepsilon }\right) \left[ \mathcal {T}_{LR}^{S}(\varepsilon )\delta \mu +\left( \frac{\varepsilon -\mu }{T}\right) \mathcal {T}_{LR}^{S}(\varepsilon )\delta T \right] . \end{aligned}$$

Introducing the bias voltage $\delta \mu =e\delta V$ the above equations can be expressed in a matrix of the form:

25 $$\begin{aligned} \left( \begin{array}{c} J_{e} \\ J_{Q} \\ \end{array} \right) = \left( \begin{array}{cc} e^2L_{00} & eL_{01} \\ eL_{10} & L_{11} \\ \end{array} \right) \left( \begin{array}{c} \delta V \\ \delta T/T \\ \end{array} \right) , \end{aligned}$$

where

26 $$\begin{aligned} L_{ij}=\frac{1}{h}\int (\varepsilon -\mu )^{i+j}\left( -\frac{\partial f}{\partial \varepsilon }\right) \mathcal {T}_{ij}(\varepsilon ) , \end{aligned}$$

with $\mathcal {T}_{00}(\varepsilon )= 2 \mathcal {T}_{LL}^{A} + \mathcal {T}_{LR}^S$, $\mathcal {T}_{01}(\varepsilon ) = \mathcal {T}_{10}(\varepsilon ) = \mathcal {T}_{11}(\varepsilon ) = \mathcal {T}_{LR}^S$. Notice that the matrix in Eq. (25) reflects the Onsager symmetry since $L_{01}=L_{10}$.

Now, let us introduce the thermoelectric coefficients describing the transport properties in the linear response regime. The charge conductance is expressed as

27 $$\begin{aligned} G=\left( \frac{J_e}{\delta V}\right) _{\delta T=0}=e^2L_{00}, \end{aligned}$$

where both sub-gap and above gap tunneling processes contribute. The Seebeck coefficient (thermopower) *S* is the ratio of the voltage $\delta V$ generated by the temperature difference $\delta T$ set between the reservoirs under the assumption of no charge current flowing through the system, $J_e=0$, so that

28 $$\begin{aligned} S=-\left( \frac{\delta V}{\delta T}\right) _{J_e=0}=\frac{1}{eT}\frac{L_{01}}{L_{00}}. \end{aligned}$$

Finally, the heat conductance, $\kappa$, is defined as the ratio of the heat current, $J_Q$, to the temperature difference, $\delta T$, assuming the absence of charge current

29 $$\begin{aligned} \kappa =\left( \frac{J_Q}{\delta T}\right) _{J_e=0}=\frac{1}{T}\left( L_{11}-\frac{L_{01}^2}{L_{00}}\right) . \end{aligned}$$

Although sub-gap states do not contribute to the heat current [Eq. (25)] in the linear response regime, they influence the heat conductance via the $L_{00}$ term in the above equation. Similarly, the Seebeck coefficient, Eq. (28), is altered by sub-gap tunneling processes, which modify the condition of vanishing charge current. To complete the linear response regime we introduce the figure of merit $ZT=GS^2T/\kappa$ which measures the thermoelectric efficiency of the system.

### Beyond linear response regime

Here, we investigate the thermoelectric properties of the considered system when the applied bias voltage and temperature difference are arbitrary large. Thus, we introduce a finite bias voltage $\Delta \mu =e\Delta V$ and a temperature difference $\Delta T$. Now, charge and heat currents are described by Eq. (21) and Eq. (22), respectively. The power extracted from the system (being generated by heat engine) is $P=-J_e\Delta V$, whereas the corresponding efficiency becomes $\eta =P/J_Q$. Here, $\Delta V$ is the voltage applied to compensate the thermally generated current $J_e$. Accordingly, the maximum power is given by $P_{max}=GV_{max}^2=\frac{1}{4}GS^2(\Delta T)^2=\frac{1}{4}G V_b^2$, whereas the efficiency at maximum power can be expressed as^125^

30 $$\begin{aligned} \eta (P_{max})=\frac{\eta _C}{2}\frac{ZT}{ZT+2}, \end{aligned}$$

where $\eta _C$ is the Carnot’s efficiency. Here, $V_b$ is the blocking voltage, i. e. the voltage which compensates the thermally generated current, $V_{max}=V_b/2$ is the voltage for which the power becomes maximal and *ZT* is calculated for the average temperature $T=(T_L+T_R)/2=T+\Delta T/2$.

## Results

In the numerical calculations we assume an asymmetry in the coupling strengths between the QD and the leads introducing dimensionless parameters $\gamma _L$ and $\gamma _R$ in the following way, $\Gamma _L=\gamma _L\Gamma$, $\Gamma _R=\gamma _R\Gamma$. Such a parametrization allows us to tune the coupling of the QD to a given lead independently of the coupling to the other one. All energy quantities are expressed in units of the zero temperature superconducting gap energy, $\Delta$. The gap values for typical superconductors are within the range $(1.5-30)\times 10^{-4}$ eV. Moreover, we assume that $\Gamma =0.1\Delta$ and we tune the coupling strength of the QD to the leads by adjusting the parameters $\gamma _\alpha$ ($\alpha =L,R$). In experiment, this can be achieved by tuning proper gate voltage which controls tunneling barrier properties.

**Figure 2:**
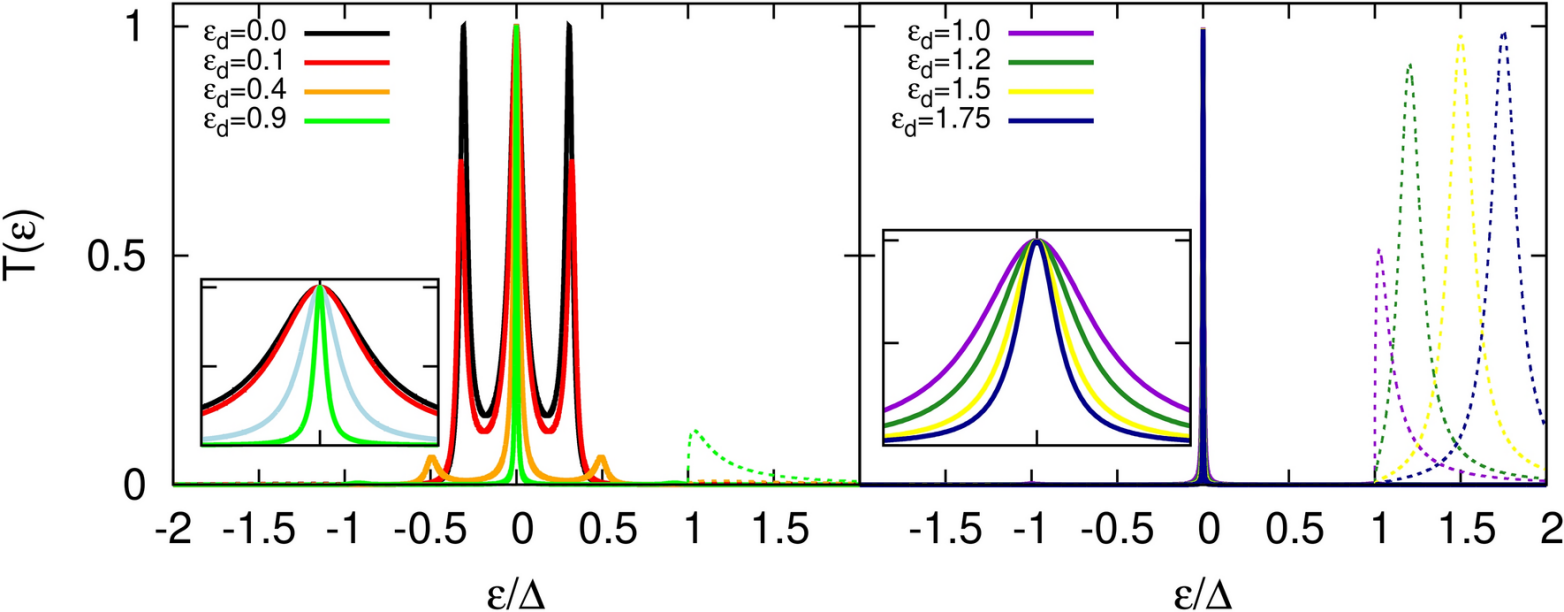
Transmission coefficients as a function of energy calculated for the indicated values of the dot’s energy level and for symmetric coupling to the leads, $\gamma _L=\gamma _R=1$. Solid lines correspond to $T_A(\varepsilon )$, whereas dotted lines are associated with $T_S(\varepsilon )$. Insets show a zoomed-in view of the central peaks region. The displayed energy range is $\varepsilon /\Delta \in [-0.1,0.1]$ for the inset in the left panel and $\varepsilon /\Delta \in [-0.01,0.01]$ for the inset in the right panel. Other parameters are: $\Gamma =0.1\Delta$, $T=0$.

### Transmission coefficients

Here, we investigate the influence of the parameters of the system on the transmission coefficients resulting from Andreev-like and quasiparticle tunneling processes, $\mathcal {T}^A_{LL} (\varepsilon ) = T_A(\varepsilon )$ and $\mathcal {T}^S_{LR} (\varepsilon ) = T_S(\varepsilon )$, respectively.

In Fig. 2 the transmission is presented for different values of the dot energy level. First of all, the transmission $T_A(\varepsilon )$ exhibits a three-peak structure due to the coupling of the dot to the MBS, in which the central peak is pinned at zero energy regardless of the energy level of the dot. Moreover, the zero energy peak (ZEP) acquires the maximal allowed intensity i. e. $T_A(\varepsilon =0)=1$ irrespective of the dot level position. The ZEP is strictly related to the zero-energy Majorana state hosted by the topological superconductor nanowire. As already mentioned, the position of the ZEP is pinned due to particle-hole symmetry. However, its width changes by tuning the energy level of the dot $\varepsilon _d$, and monotonously decreases when increasing $\varepsilon _d$. When the dot level energy is close to zero, the width of the ZEP is mainly due to the broadening caused by the coupling of the dot to the normal lead. However, when $\varepsilon _d$ moves away from zero energy, the ZEP shrinks and finally for $\varepsilon _d\rightarrow \infty$ the ZEP becomes totally localized and its width vanishes. The dot level position dependence of the ZEP in the considered system is in total opposition to that of Andreev peaks for a QD coupled to a normal metal and an s-wave superconductor. In the latter case, increasing the dot’s energy level leads to Andreev peaks moving away and to a decrease of the conductance. In the former case, the change in the dot energy level does not influence the position nor the intensity of the ZEP, which has profound consequences on the conductance (as shown later).

When the dot level is tuned to zero energy, the transmission $T_A(\varepsilon )$ exhibits two side-peaks, on top of the ZEP, of maximal intensity i. e. equal to the intensity of the central one. These peaks are a consequence of the proximity effect with the topological superconductor via the self-energy term $~1/\varepsilon$. Note that this term is not constricted to the sub-gap energy range. However, when tuning the dot level position away from zero energy, these peaks start to vanish and their positions spread apart. This resembles the dot level dependence of Andreev peaks in a normal metal / QD / s-wave superconductor junction. Here, the position of the side-peaks is a complex function of the QD couplings and the dot energy level. As can be seen, for $\varepsilon _d\approx 0.4\Delta$ only small features corresponding to these states can be noticed in $T_A(\varepsilon )$ and with further increase of the dot level position the maxima practically disappear. Indeed, they still exist but acquire vanishingly small intensities and move away from the central peak as $\varepsilon _d$ grows. Interestingly, with further increase of dot energy level, $|\varepsilon _d|>\Delta$, these peaks leak into the above gap region (which is not surprising as the corresponding self-energy is not restricted to the sub-gap region) unlike conventional Andreev peaks which are confined to the sub-gap region, i. e. for $|\varepsilon _d|>\Delta$ the Andreev peaks do not leak above the gap and stay located at energies close to $\pm \Delta$. For comparison, see also Fig. 6 in Supplementary Information, where transmission coefficient for NM-QD-(*s-wave*)SC is presented.

The sub-gap peaks in $T_A(\varepsilon )$ are symmetric with respect to zero energy regardless of the dot energy level, which is a consequence of the particle-hole symmetry. However, this does not hold for above gap transmission, $T_S(\varepsilon )$. Indeed, while for $\varepsilon _d=0$ the transmission $T_S(\varepsilon )$ exhibits small features for energies $|\varepsilon |>\Delta$ which are symmetric with respect to zero energy, these structures become asymmetric as the dot energy level moves away from zero. For $\varepsilon _d>0$, as one increases the dot energy level, the structure at energy $\varepsilon >\Delta$ gets more pronounced, while the one at $\varepsilon <-\Delta$ looses in intensity. The opposite occurs for negative dot energy level. When the dot energy level is above the gap, $\varepsilon _d\ge \Delta$, the above gap transmission $T_S(\varepsilon )$ becomes significant for $\varepsilon >\Delta$. The intensity of this peak in $T_S(\varepsilon )$ grows proportionally to the TS’s density of states when increasing the dot’s level position, signaling that the quasiparticle tunneling starts to dominate transport. Finally, for $\varepsilon _d\gg \Delta$ the intensity of the peak in $T_S(\varepsilon )$ saturates, achieving the unitary limit.

**Figure 3:**
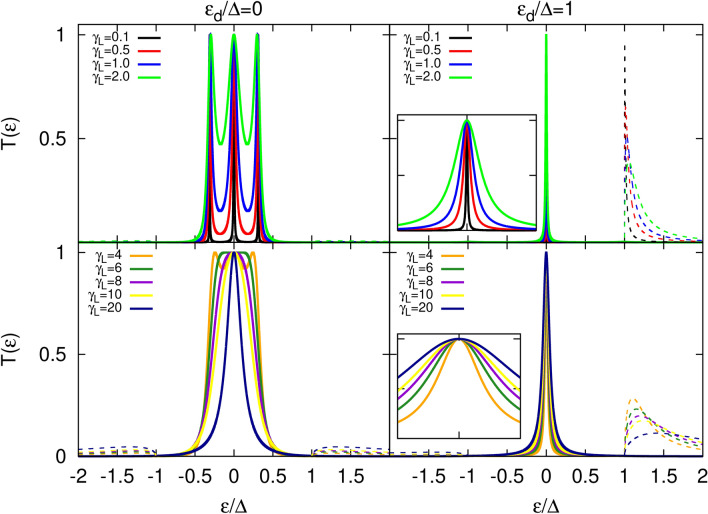
Transmission coefficients as a function of energy calculated for indicated values of the parameter $\gamma _L$, measuring the QD’s coupling strength to the left lead, and for constant coupling of the dot to the right electrode, $\gamma _R=1$. Left panel shows results for $\varepsilon _d=0$ (dot’s energy level located within the SC energy gap), whereas the right panel corresponds to a dot’s energy level at the boundary of the SC gap, $\varepsilon _d=\Delta$. Solid lines correspond to $T_A(\varepsilon )$, whereas dashed lines are associated with $T_S(\varepsilon )$. Insets show a zoomed-in view of the central peaks region. For better comparison, the energy range in both insets is the same, $\varepsilon /\Delta \in [-0.04,0.04]$. Other parameters are the same as in Fig. 2.

**Figure 4:**
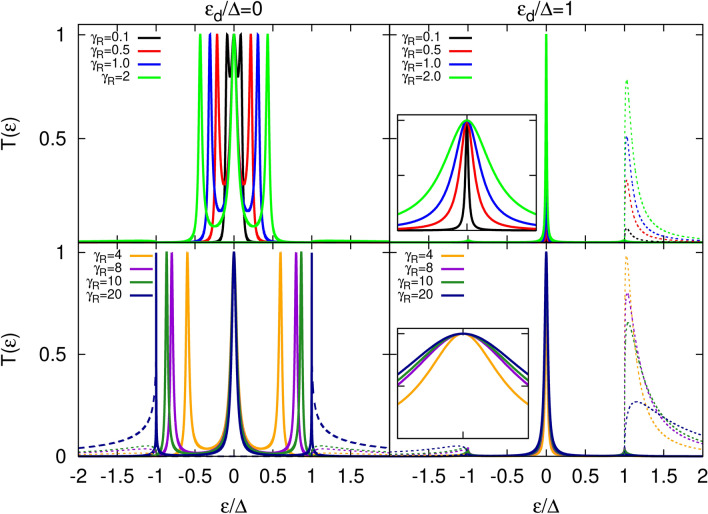
Transmission coefficient as a function of energy calculated for indicated values of the parameter $\gamma _R$ and for a given $\gamma _L=1$. The left panel shows results for $\varepsilon _d=0$, whereas the right panel corresponds to $\varepsilon _d=\Delta$. Dashed lines correspond to $T_A(\varepsilon )$, whereas solid lines are associated with $T_S(\varepsilon )$. Insets show a zoomed-in view of the central peaks region. For better comparison, the energy range in both insets is the same, $\varepsilon \in [-0.02,0.02]$. Other parameters are the same as in Fig. 2.

We discuss now the influence of the dot coupling strength to the normal metal (left) and topological superconductor (right) reservoirs. In Fig. 3 we show the transmission coefficients, $T_A(\varepsilon )$ and $T_S(\varepsilon )$, for various values of the dot coupling to the left lead (normal metal), keeping constant the coupling to the right electrode. We consider two distinct cases: when the dot energy level is located inside the energy gap of the superconductor (left panel) and when it lies at the edge of the TS gap (right panel). Let us first discuss the case when $\varepsilon _d=0$. For sufficiently small coupling to the left electrode, there are three well-resolved peaks in the transmission coefficient $T_A(\varepsilon )$. While increasing the coupling strength of the dot to the left electrode, these peaks get broadened, ultimately merging into one single peak for sufficiently large coupling strength. However, this single peak then shrinks with further increase of the parameter $\gamma _L$. Simultaneously, small features in $T_S(\varepsilon )$ for energies $|\varepsilon |>\Delta$ become visible. The shrinking of the central peak starts to occur when the dot level width extends beyond the SC gap and begins to overlap with the quasiparticle states. In turn, for a dot energy level outside the gap ($\varepsilon _d\ge \Delta$) the width of the central peak in $T_A(\varepsilon )$ monotonically grows with increasing $\gamma _L$ which results in the growing tail corresponding to the dot level state. The change in coupling to the left lead still does not influence the intensity of this peak, which stays maximal. The above gap feature in $T_S(\varepsilon )$, however, is more pronounced than in the resonant situation considered before ($\varepsilon _d=0$). When increasing the coupling strength to the left lead, this structure gets smeared over a broader range of energy, with an intensity which drops significantly.

In Fig. 4 we show the transmission coefficients for various values of the dot’s couplings to the right electrode (topological superconductor) keeping constant the coupling to the left reservoir. As in the previous situation, we consider two cases i. e. when the dot energy level is situated inside the energy gap of the TS (left panel) and when it lies at the edge of the gap (right panel). For the former case, increasing the coupling parameter $\gamma _R$ leads to the side-peaks in $T_A(\varepsilon )$ moving away from the central resonance without changing their intensity. Simultaneously, the transmission $T_S(\varepsilon )$ outside the superconducting gap is only slightly enhanced for $\gamma _R\le 10$ before increasing substantially for sufficiently large value of the coupling parameter. Note also, that tuning $\gamma _R$ does not lead to leaking of the satellite-peaks of $T_A(\varepsilon )$ into the above-gap region as for sufficiently large coupling to the TS reservoir these maxima are pinned at $\pm \Delta$ and still possess maximal intensity. As long as the side-peaks are located well inside the gap, their width is insensitive to the change in $\gamma _R$. For sufficiently large values of the coupling, these maxima move close to the SC gap edges where they shrink greatly. In turn, in the later case ($\varepsilon _d\ge \Delta$), the subgap energy region hosts only a central peak in $T_A(\varepsilon )$ which broadens as one increases the coupling strength $\gamma _R$. More importantly, tuning the coupling to the right electrode leads to structures in $T_S(\varepsilon )$ at energies outside the gap, which get broader as $\gamma _R$ increases. While the feature at negative energies is slowly enhanced, the one at positive energy evolves non-monotonically with $\gamma _R$. There, the unitary limit is reached for $\gamma _R=4$ and a further increase of the coupling to the TS reservoir leads to a reduction of the amplitude of this structure in $T_S(\varepsilon )$.

### Thermoelectricity in linear response regime

Now, that we understand how the transmission coefficients behave as a function of energy, we can use these results to compute the transport properties and explain the features we observe there. Thus, in this section we consider the thermoelectric response of the system in the linear response regime. Particularly, we study quantities, including the electric conductance, the Seebeck coefficient, the heat conductance and the corresponding figure of merit, defined in Linear response regime. Here, we assume a temperature dependence of the superconducting gap as

31 $$\begin{aligned} \Delta (T)=\Delta \sqrt{1-(T/T_c)^3} \end{aligned}$$

with $\Delta$ denoting the energy gap at zero temperature and $T_c$ being the critical temperature, at which superconductivity vanishes. Next, we assume that $T_c$ is coupled to $\Delta$ by the BCS equation, $\Delta =1.764k_BT_c$. For typical superconductors $T_c$ ranges from 1 K to 20 K. In the case of a nanowire with strong spin-orbit interactions being in proximity to an s-*wave* superconductor and placed in a magnetic field enabling the transition to a topological phase, the gap function should inherit the properties of the s-*wave* superconductor, and we thus expect the above relation to be fulfilled. In a more general case, the relation between $\Delta$ and $T_c$ is given by $\Delta =1.764\exp {(-\langle |g_\textbf{k}|^2\ln {(g_\textbf{k})}\rangle _{\textbf{k},FS})}k_BT_c$ with $g_\textbf{k}$ denoting the gap anisotropy function. For a s-wave SC the gap function is isotropic and is given by $g_k=1$; for a p-wave SC $g_k$ is anisotropic and depends on the two-dimensional momentum vector $(k_x+ik_y)/|k|=\exp {(i\phi )}$ with the angle $\phi =\arctan {(k_y/k_x)}$. A similar equation should be valid for a p-*wave* superconductor described by the pairing potential $\Delta (\phi )=|\Delta |e^{i\phi }$, denoting the phase $\phi$. Moreover, for point-like impurity one has $\phi =0$ which covers the case of topological superconductor wires^126^.

**Figure 5:**
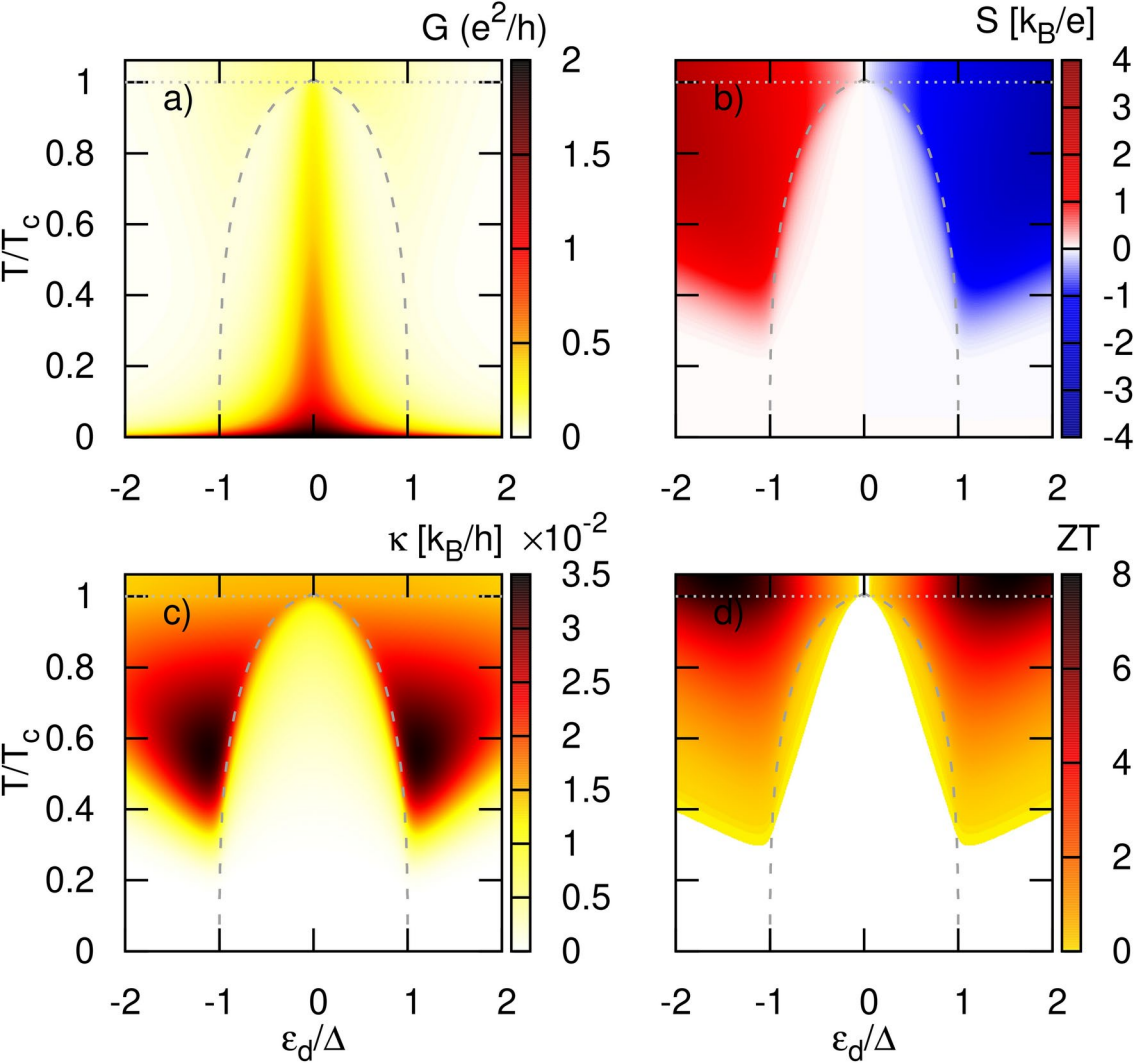
Thermoelectric coefficients: (**a**) electrical conductance, (**b**) Seebeck coefficient (thermopower), (**c**) heat conductance, (**d**) figure of merit, calculated as a function of the dot’s energy level $\varepsilon _d$ and temperature $k_B T$ for $\gamma _R=\gamma _L=1$ and $\Gamma =0.1\Delta$. The dotted gray horizontal line indicates the critical temperature $T_c$ at which the energy gap of the superconducting lead vanishes. The dashed gray line shows the temperature dependence of the superconducting energy gap $\Delta (T)$. Here and in the next figures $e=-|e|$.

In Fig. 5 we present the thermoelectric coefficients as a function of the dot energy level, $\varepsilon _d$, and temperature, $k_BT$, for symmetric couplings ($\gamma _L=\gamma _R$). The gray dashed line indicates the superconducting gap given by Eq. (31), whereas the gray dotted line shows the critical temperature $k_BT_c$.

First of all, the conductance is shown in Fig. 5a. At low temperature, $k_BT\ll \Delta$, the main contribution to the conductance in the linear limit comes from the tunneling through sub-gap states yielding a central peak. As the temperature is increased, quasiparticles become activated and start to play a more prominent role in the transport at higher values of $\varepsilon _d>\Delta (T)$.

The most prominent feature is the central subgap peak centered in $\varepsilon _d=0$ which is associated with the zero-energy Majorana bound state. At very low temperature, the electrical conductance reaches the quantum limit and its intensity is practically unchanged over a relatively broad range of the dot energy level $\varepsilon _d$. This is a direct consequence of the behavior of the transmission coefficient $T_A$ described in the previous section (see also Fig. 2). The Majorana peak in transmission is pinned to zero energy which leads to a maximum conductance at low temperature regardless of the dot level position i. e. for temperature $T\rightarrow 0$ the conductance is given by $G=2e^2/h T_A (\varepsilon =0)=2e^2/h$ because $T_A(\varepsilon =0)=1$. A similar behavior has been predicted in QD systems coupled to two normal metal leads and connected to a Majorana nanowire^100,101^. When the temperature is increased the intensity and the width of the Majorana-related peak in conductance shrinks, ultimately vanishing at the critical temperature $T=T_c$.

As already mentioned, both subgap and above gap tunneling processes contribute to the electrical conductance outside the superconducting gap, i. e. for $|\varepsilon _d|>\Delta (T)$ (a situation which is in stark contrast with a conventional s-*wave* SC electrode where the contribution from Andreev reflection processes is limited to the subgap conductance). These two contributions compete with each other leading to a non-monotonic temperature dependence of the total electrical conductance. At low temperature, *G* is dominated by tunneling processes through the tails of the ZEP. This contribution is maximal at zero temperature, then decreases monotonically with *T*, leading to a rapid drop in the electrical conductance. Increasing the temperature further then favors quasiparticle tunneling processes, whose contribution increases monotonically with *T* up to $k_BT=\Delta (T)$ Consequently, the electrical conductance reveals a characteristic minimum at certain temperatures depending on the dot level position. We point out that this phenomenon is absent in a system where the TS is replaced by a conventional s-*wave* SC electrode since Andreev processes are fully suppressed outside the gap. Finally, the electrical conductance reaches a second maximum for higher temperatures, in the normal state ($T>T_c$), before slowly decreasing due to the Fermi-Dirac temperature dependence.

In Fig. 5b the Seebeck coefficient (thermopower) is presented as a function of both the dot energy level and the temperature. One notices that the thermopower is strongly suppressed (practically zero) inside the gap which results from particle-hole symmetry as it ensures that subgap states (zero-energy MBS and satellite peaks) do not contribute to the charge current in the presence of a temperature difference. The only remaining contribution arises from quasiparticle tunneling, which is heavily suppressed within the superconducting gap, and only gives a small contribution to the thermopower in the vicinity of the gap edges, ie for $\Delta -\Gamma _L\lesssim |\varepsilon _d|<\Delta$ (a consequence of the dot level broadening being proportional to $\Gamma _L$).

When $\varepsilon _d$ lies outside the gap, the Seebeck coefficient increases rapidly as quasiparticle tunneling becomes allowed and contributes to the charge current. To compensate the charge current induced by the temperature difference one needs to apply a finite voltage which results in a nonzero thermopower. The thermopower is such that $S>0$ above the superconducting gap and the transport is electron-like, whereas below the gap $S<0$ and the transport is hole-like. Interestingly, one notices that at low temperature, the thermopower is strongly attenuated outside the gap [obscured in the Fig. 5b]. Indeed, quasiparticle tunneling requires some energy to be activated and to participate in the charge and heat transfers. For low temperature, the number of quasiparticles involved in transport is very small which results in infrequent transfer of energy and charge via the transmission $T_S$. Simultaneously, the electrical conductance is still sizable (due to Andreev processes through the transmission $T_A$, as argued above) so that the resulting Seebeck coefficient is strongly suppressed. For sufficiently large temperature, however, the absolute value of the thermopower |*S*| attains relatively large values as quasiparticle tunneling becomes dominant. At fixed temperature, the thermopower also increases with increasing dot energy level reaching a (temperature-dependent) maximum before slowly decreasing back as $|\varepsilon _d|$ further increases. This enhancement of the thermopower is a consequence of the small electrical conductance obtained for large values of $|\varepsilon _d|$, as this implies that the external voltage applied to compensate the thermally induced current must be relatively large (as can be inferred from the definition of *S* in Eq. (28) where the denominator is  *G*). It is worth pointing out that the temperature dependence of the Seebeck coefficient differs from that of the normal metal / QD / s-*wave* superconductor junction^56^. Indeed, in that case, some finite thermopower leaked into the gap for sufficiently large temperature (but still lower than $T_c$)^56,84^. This can be attributed to the different behavior of the quasiparticle density of states, as in the s-*wave* SC case, the density of states diverges at $|\varepsilon |=\Delta$, yielding a greater contribution to *S* even when the dot energy level lies deep inside the SC gap. The same difference in density of states for the TS and s-*wave* superconductors also explains the difference in behavior for $|\varepsilon _d|>\Delta$. For comparison see Fig. 7 and Fig. 9 in the Supplementary Information.

The thermal conductance, $\kappa$, is shown in Fig. 5c. In the linear response regime, only quasiparticle tunneling contributes to the heat current (see Eq. (24)) [more generally, this is true as long as the system is only thermally biased, see Eq. (22)]. Thus, the heat conductance is only residual within the superconducting gap and reaches the largest values outside the gap. For a fixed temperature, it shows two maxima corresponding to quasiparticle tunneling to states above/below the gap in the topological superconductor. Although single electrons carry a relatively large energy, $\varepsilon \sim \Delta$, the heat conductance is small at low temperature because quasiparticle tunneling is strongly suppressed. As the temperature increases, the quasiparticle tunneling processes become activated which results in a larger heat conductance, reaching a global maximum for $k_BT/\Delta \approx 0.3$ and $|\varepsilon _d|/\Delta \approx 1.2$. Increasing the temperature further leads to a slow decrease of the heat conductance resulting from the temperature dependence of the Fermi-Dirac distribution: while there are more high energy electrons excited in the normal metal lead, there are also less available quasiparticle states in the TS electrode to which these electrons can tunnel.

It is worth noting that although the subgap states (associated with the tunneling term $T_A$), which include the Majorana bound state, do not contribute to the heat transfer into the topological superconductor in a purely thermally biased system, they do influence the heat conductance. Indeed, the latter is defined at the condition of zero charge current, which contains contributions from both $T_S$ and $T_A$. In the low temperature regime, the electrical conductance is mainly associated with the ZEP, i. e. the $T_A$ term, and as there is not enough energy to excite quasiparticles lying above the gap, the heat transfer is suppressed, yielding only a residual heat conductance. For higher temperature, quasiparticle tunneling starts to dominate electrical transport and the energy transfer also increases. This behavior, together with the temperature dependence of the Fermi-Dirac distribution, leads to a non-monotonic temperature dependence of the total electrical conductance, as previously explained. In turn, the behavior of *G* changes the condition of vanishing charge current under which the heat conductance is determined. In particular, the $T_A$ contribution to *G*, and thus, to $\kappa$ through the $L_{00}$ term, enhances the heat conductance for a wide range of temperature below $T_c$.

Again, the behavior of the present junction departs from its s-*wave* SC counterpart when it comes to the heat conductance. Indeed, while in the TS case, the heat conductance is strongly suppressed deep inside the SC gap and may only reach significant values in the vicinity of the gap edges, in the conventional SC case, the heat conductance may achieve relatively large values deep in the SC gap, even higher than outside the gap^56,84^. For detailed comparison see also Supplementary Information. In turn, in the case of the TS system at fixed temperature, the heat conductance inside the gap is always smaller than that outside the gap, regardless of the temperature.

The above described dependence of the Seebeck coefficient *S* along with the electrical and heat conductances *G*,$\kappa$, determines the figure of merit *ZT* shown in Fig. 5d. Here, we neglected the contribution of the lattice to the heat conductance. First, the figure of merit is strongly suppressed deep inside the superconducting gap which is the result of a vanishingly small thermopower for $|\varepsilon _d|<\Delta$. It reaches relatively small but finite values in the vicinity of the superconducting gap edges, as was already noticed for the thermopower (a consequence of the the dot level broadening). At fixed temperature, the figure of merit increases when increasing $|\varepsilon _d|$, reaching maxima outside the SC gap before further decaying due to the suppressed electrical conductance. This decay remains rather slow due to the reduction of the heat conductance concomitantly with the increase in the (absolute value of the) Seebeck coefficient. Interestingly, *ZT* can achieve large values right below the critical temperature i. e. $ZT\approx 7$. However, the global maxima of *ZT* are found in the normal phase, where $ZT\approx 8$ for the assumed parameters. Varying the coupling of the dot to the electrodes can further influence the thermoelectric response. In particular, the electrical conductance exhibits a noticeable broadening of the central peak, while the other thermoelectric coefficients change more quantitatively. However, for larger asymmetry parameter $\gamma _R$, both *S* and $\kappa$ *leak* deeper into the superconducting gap. Stronger coupling to the TS electrode leads to higher quasiparticle tunneling rates, resulting in greater energy transfer, which explains the enhancement of $\kappa$ even deep within the superconducting gap. For more details, refer to the Supplementary Information.

### Heat engine-power and its efficiency

The system can work as a nanoscale thermoelectric heat engine when an external load is attached to the system. The output power, $P=-J_e V$, can be extracted when a bias voltage *V* is applied against which the thermoelectric current can do work. The corresponding efficiency is given by $\eta =P/J_Q$ (see Beyond linear response regime section).

**Figure 6:**
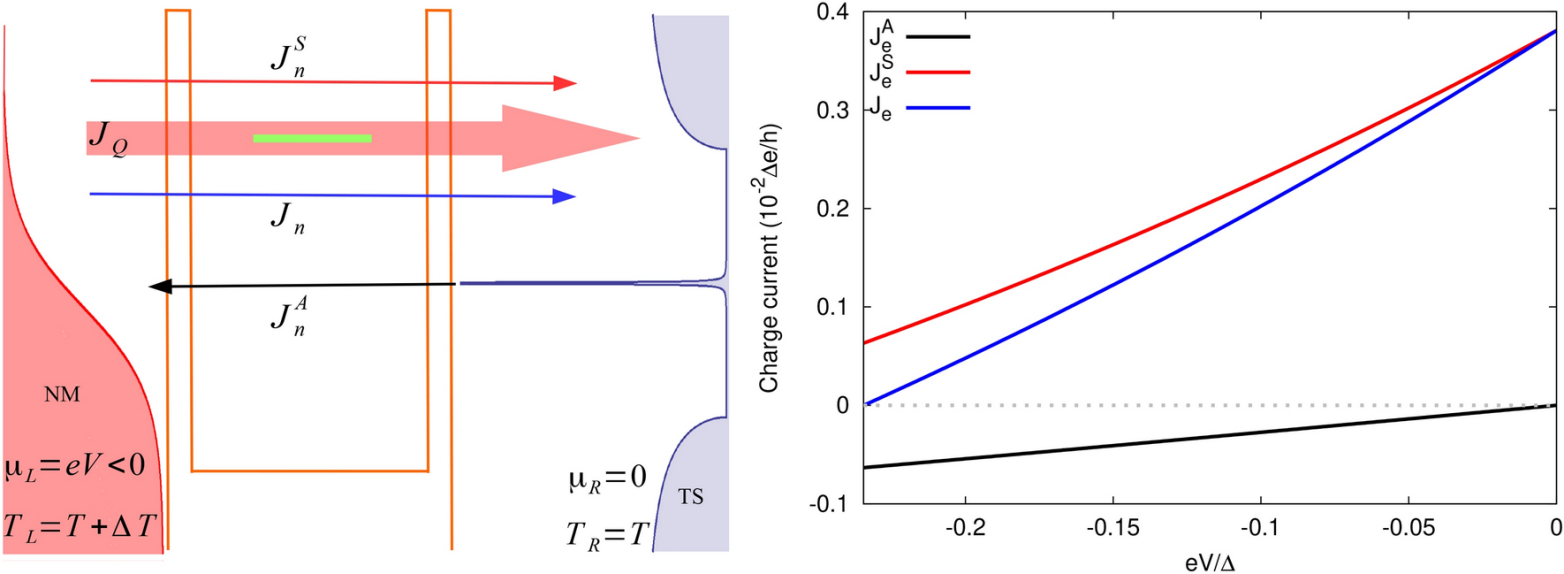
Schematic explanation of the processes described in the text (left panel) and contributions to total charge current $J_e$: $J_e^A$ and $J_e^S$ (right panel) calculated for $\varepsilon _d=1.2\Delta$. This situation holds for $\varepsilon _d\ge \Delta$. In the scheme thick red arrow shows the direction of heat current, whereas thin arrows denote the direction of sub-gap ($J_n^A$), QP ($J_n^S$) and total ($J_n$) particle current when positive temperature difference ($\Delta T>0$) and negative bias voltage ($eV<0$) is applied. Particle current is simply related to charge current by $J_e/e$ and similarly for $J_n^A$ and $J_n^S$.

Although, in the non-equilibrium regime the heat current due to sub-gap tunneling processes is finite [see Eq. (22)] and directly related to the relevant charge current, $J_{Q}^{A}=-V J_{e}^{A}\equiv -eV J_{n}^{A}$, it does not result in useful output power. This is related to the fact that the temperature difference set to the system is not able to generate a charge current $J_{e}^{A}$ as particle-hole symmetry holds (as explained in Thermoelectricity in linear response regime). On the other hand, the leaking current into the SC gap resulting from quasiparticle tunneling is strongly suppressed. In turn, the applied voltage drives both types of currents, from which the one associated with $J_{e}^{A}$ is dominant for $|\varepsilon _d|\ll \Delta (T)$. Thus, it is enough to apply a small bias voltage to compensate for the thermally induced current in this regime. Interestingly, the charge current $J_{e}^{A}$ flows opposite to the quasiparticle current in the whole operational range. Consequently, the heat current resulting from sub-gap tunneling processes (and generated by bias voltage) flows in the opposite direction than the temperature gradient ($\delta T=T_L-T_R$) driving the charge current. As a result, charge current, $J_{e}^{A}$, heats the reservoir with higher temperature. Of course, the total current, for the bias voltage range for which the power is finite, extracts the heat from the hot lead. Figure 6 schematically explains the above process. Note that total particle current flows in the same direction as the net heat current, whereas corresponding total charge current is opposite to both of them. Heat current contributions, $J_Q^A$ and $J_Q^S$, flow in the same directions as corresponding particle currents. Negative bias voltage *eV* applied tries to compensate the thermally generated thermocurrent (for $eV=0$ finite current is flowing due to temperature difference $\Delta T$) leading to suppression of $J_n^S$ and $J_n$ and simultaneously to enhancement of $|J_n^A|$. $J_n^A$ doesn’t contribute to thermocurrent ($J_n^A=0$ for $eV=0$) but is driven by applied bias voltage and its direction is opposite to both $J_n^S$ and $J_n$. Thus, it takes part in compensation of thermally generated thermocurrent. The thermocurrent becomes totally blocked for bias voltage achieving blocking voltage value.

**Figure 7:**
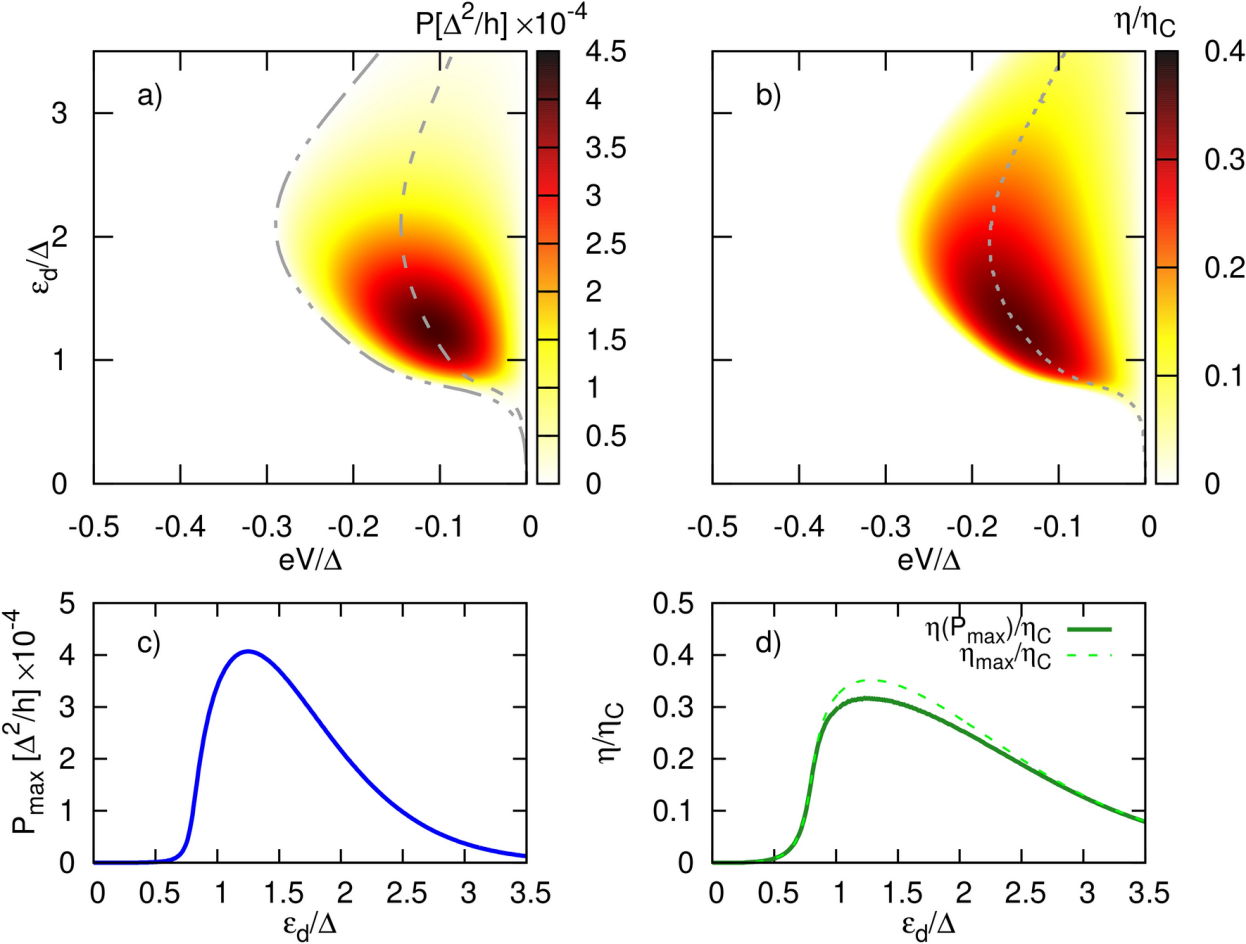
Bias voltage and dot energy level dependence of (**a**) the output power, (**b**) the corresponding thermal efficiency normalized to Carnot’s efficiency, calculated for $k_BT=0.4\Delta$, $\Delta T=0.1\Delta /k_B$. The dashed gray line in (**a**) [(**b**)] indicates the maximum power [maximum efficiency], whereas the dash-dotted gray line in (**a**) indicates the blocking voltage. Corresponding maximum power (**c**), efficiency at maximum power and maximum efficiency (**d**) as a function of the dot level. Other parameters: $\gamma _L=\gamma _R=1$ and $\Gamma =0.1\Delta$.

In Fig. 7 we present the output power [Fig. 7a] and the corresponding efficiency [Fig. 7b] as a function of the applied bias voltage *eV* and the dot energy level $\varepsilon _d$ calculated for indicated values of the temperature and for symmetric couplings, $\gamma _L=\gamma _R=1$. The lower left panel shows the maximum power [Fig. 7c] as a function of the dot energy level extracted from Fig. 7a, whereas Fig. 7d presents both the efficiency at maximum power ($\eta (P_{max})$) and the maximum efficiency ($\eta _{max}$) as a function of the dot energy level. The gray dashed line in Fig. 7a indicates the maximum power which is shown in Fig. 7c, whereas the gray dash-dotted line denotes the blocking voltage i. e. the voltage at which the thermally generated current is compensated for by the applied bias voltage. This line has been obtained self-consistently by imposing the condition, $J_L^e=0$, whereas the line corresponding to maximum power is related to the blocking voltage by the simple equation $V_{max}=V_b/2$. Moreover, the gray dashed line in Fig. 7b denotes the maximum efficiency which has been extracted from the numerical data. Note that, in general, the maximum efficiency does not correspond to the efficiency at maximum power, which is clearly seen in Fig. 7d.

The output power grows with increasing *eV* until it reaches a maximum. It then decreases with a further increase in *eV*, and finally reaches zero at voltage $V = V_b$, at which the thermally generated current becomes totally compensated by the current induced due to the applied bias voltage. This blocking voltage thus corresponds to the Seebeck voltage induced by a difference in temperature under open circuit condition. One notices that the power can be extracted only for $V\in [0,V_b]$. The corresponding efficiency, normalized by Carnot efficiency $\eta _C$, and shown in Fig. 7b, roughly follows the output power dependence. Comparing the maxima of Fig. 7a,b readily confirms that maximum efficiency does not mean maximum power, as usually maximum power is achieved with non-maximum efficiency. Moreover, the power is strongly suppressed for dot’s energy level situated inside the SC gap. i. e. for $|\varepsilon _d|<\Delta (T)$, which is direct consequence of the vanishingly small quasiparticle current inside the gap (as explained earlier).

Tuning the dot energy level and applying the relevant bias voltage one can extract the maximal allowed power for a given set of parameters of the system. Indeed, from the application point of view, the most important quantities are the maximum power and the corresponding efficiency (at maximum power). These are presented in the lower panels of Fig. 7 as a function of the dot energy level. Fig. 7d clearly shows that the efficiency at maximum power is never larger than maximum efficiency. From Fig. 7c,d, it can be inferred that maximum of $P_{max}$ and $\eta _{P_{max}}$ correspond to the same value of the dot energy level. Moreover, the maximal value of $\eta _{P_{max}}$ corresponds to $ZT\approx 3.6$ which indicates that the system can be utilized as a good heat to electrical power converter. This value has been obtained from inverting Eq. (30) and substituting $\eta _{P_{max}}\approx 0.32\eta _C$ corresponding to $\varepsilon _d\approx 1.23\Delta$. Interestingly, $\eta _{P_{max}}$ and $\eta _{max}$ acquire the same values for $|\varepsilon _d|<\Delta (T)$ and also for sufficiently large dot level positions.

**Figure 8:**
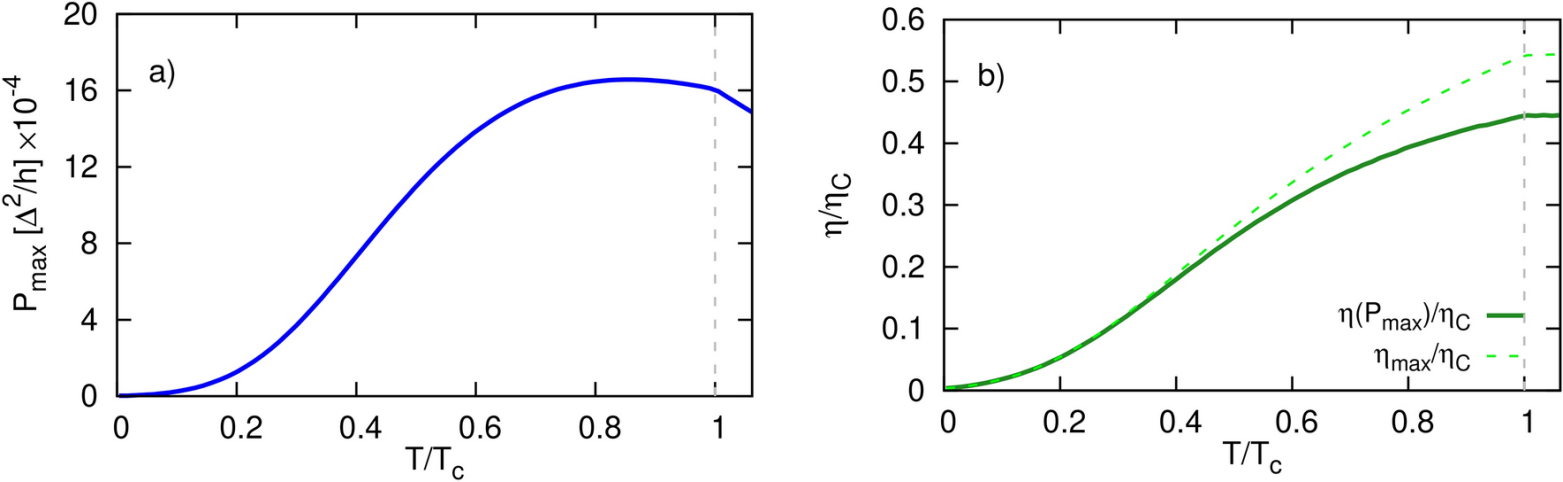
Temperature dependence of (**a**) the maximum power, (**b**) the corresponding efficiency at maximum power (solid green line) and maximum efficiency (dashed green line), calculated for $\varepsilon _d=1.2\Delta$, $\Delta T=0.2\Delta /k_B$. The dashed gray line indicates the critical temperature. Other parameters: $\gamma _L=\gamma _R=1$ and $\Gamma =0.1\Delta$.

The extracted power and the corresponding efficiency can be further optimized by varying the various parameters of the system including the ambient temperature, the temperature difference set between the electrodes, and the couplings strength of the dot to the leads. The relevant results are shown in Supplementary Information and here we only briefly list the most interesting features. Both the increase of temperature *T* and/or the temperature difference $\Delta T$ leads to an enhancement of the maximum power, maximum efficiency and the efficiency at maximum power. Specifically, $\eta _{max}$ and $\eta (P_{max})$ grow monotonically with increasing temperature up to $T=T_c$, and above $T_c$ are almost saturated in the presented range for $T>T_c$ (see Fig. 8 and Fig. 3 in SI). Simultaneously $P_{max}$ increases achieving maximum for $T_{max}\approx 0.85T_c$, then drops when further increasing *T*. However, the respective drop of $P_{max}$ for $T>T_c$ is much faster than that observed for $T_{max}<T<T_c$. This clearly indicates that greater power can be extracted in the SC phase than in the normal phase with quite similar efficiency, approximately equal to $0.45\eta _C$. Note that $\eta (P_{max})/\eta _C$ for the presented range of $T\ge T_C$ is roughly constant. For the temperature range, $k_BT<0.4 T_c$, one notices that $\eta _{max}=\eta (P_{max})$ for $\Delta T=0.2\Delta /k_B$.

The operation of the heat engine can also be optimized by tuning the coupling strength of the dot to the normal electrode, while keeping the coupling to the TS reservoir unchanged, as presented in Fig. 4 of the SI. These results show that the best performance can be achieved for $\gamma _L$ being around 0.7, whereas for $\gamma _L>1$ the heat engine ceases to work effectively as the resulting $ZT<1$. Finally, Fig. 5. of SI presents the operational performance of the device when the coupling strength to the TS reservoir is changed while keeping constant the one to the normal metal electrode. Although the maximum power reaches relatively large values for a broad range of $\gamma _R$, the corresponding efficiencies are rather small. The maximum power is achieved for moderate values of $\gamma _R$, i. e. for $\gamma _R\approx 2$, however, the corresponding ZT is only around 0.63.

Here we have shown the results obtained for positive dot energy level, however, the same picture is valid for negative values of $\varepsilon _d$ when reverting the bias voltage, $eV\rightarrow -eV$. Notice that only the current due to quasiparticle tunneling can do work as the power is relatively large/finite only for $|\varepsilon _d|\ge \Delta (T)$, whereas for $|\varepsilon _d|<\Delta (T)$ the output power is suppressed. More precisely, it is finite only for an extremely small range of applied bias voltage for $|\varepsilon _d|<\Delta (T)$. Note that the gray dash-dotted line in Fig. 7 constricts the region of the finite output power, whereas outside of it, the device cannot work as a heat engine.

## Conclusions

In conclusion, we have investigated the thermoelectric response of a quantum dot coupled to a normal metallic electrode and attached to a topological superconductor both in equilibrium and non-equilibrium situation. Using the Green’s function technique, we have determined electrical and heat currents flowing through the system. We also derived relevant thermoelectric coefficients valid in the linear response regime. Although the main focus was on the thermoelectric properties, we began our studies by analyzing the transmission coefficients responsible for Andreev-like and above gap tunneling processes for better understanding of the forthcoming results. We have shown that the central peak in the transmission related to the Majorana bound state is pinned at zero energy and acquires maximal intensity regardless of changes in the systems parameters like e. g. dot’s level energy, dot’s coupling to metal or TS leads. On the other hand, the satellite transmission peaks associated with Andreev-like processes turned out to not present such immunity if the dot level is moved away from zero energy. In turn, the transmission coefficient corresponding to the above gap processes becomes relevant only for dot levels being located outside the SC energy gap, and strongly depends on the variation of the coupling strengths to the normal metal and the TS leads.

It was shown that quasiparticle tunneling processes to the states above or below the superconducting gap determine the pronounced thermoelectric response of the system, whereas Andreev-like tunneling generates neither thermopower nor heat current. However, these processes contribute to the electrical conductance and influence the thermoelectric coefficients, like the thermopower and the heat conductance, through the condition of vanishing charge current at which they are defined. Although, thermopower, heat conductance and the resulting figure of merit are suppressed within the SC gap due to particle-hole symmetry, some *leakage* of thermopower and heat conductance into the SC gap can be noticed, especially, near the SC gap edges. It was shown that the *leakage* can be boosted by increasing the coupling to the TS electrode. The thermopower and the figure of merit reach quite remarkable values out of the SC gap, both in the SC and normal phases. However, when the system works as a heat engine, the extracted maximum power is greater for the SC phase than for the normal phase, e, while the corresponding efficiencies are comparable. This indicates that NM/QD/TS allows for better heat to work conversion in the SC phase. Additionally, it was shown that a proper tuning of the parameters of the system can optimize heat to useful power conversion.

## Supplementary Information

Supplementary Information.

## Data Availability

The datasets used and/or analysed during the current study available from the corresponding author on reasonable request.
