# Supplementary material for: Thermoelectric properties of a quantum dot attached to normal metal and topological superconductor
Source: Sci Rep. 2025 Jan 24;15:3068. doi: 10.1038/s41598-024-84770-w (PMC11760902; doi:10.1038/s41598-024-84770-w)
Supplement: Supplementary file 1 — Supplementary Information. [file 41598_2024_84770_MOESM1_ESM.pdf]

Thermoelectric properties of a quantum dot attached to normal metal and topological superconductor

Piotr Trocha[1,2,*], **Thibaut Jonckheere**[2], **Jérôme Rech**[2], **and Thierry Martin**[2]

[1]Institute of Spintronics and Quantum Information, Faculty of Physics and Astronomy, Adam Mickiewicz University, Poznań, 61-614, Poland
[*]ptrocha@amu.edu.pl
[2]Aix Marseille Université, Université de Toulon, CNRS, CPT UMR 7332, 13288, Marseille, France

Thermoelectricity – γ_R-dependence

Let us now briefly turn to the behavior of the thermoelectric coefficients when varying the couplings of the dot to the electrodes. In Fig. 1 the thermoelectric coefficients are presented for a relatively large asymmetry in the couplings to the left and right leads. More specifically, the coupling of the QD to the topological superconductor is ten times larger than that with the normal lead, $\gamma_R = 10\gamma_L$. Generally, the results are very similar to those obtained in the symmetric case. The only major qualitative difference can be seen in the electrical conductance with a noticeable broadening of the central peak, whereas the other thermoelectric coefficients vary only quantitatively. Indeed, the broadening of the central peak in the electrical conductance is due to the behavior of the transmission coefficient T_A presented in Fig. 4 of the main text. For ε_d close to zero, the increase of the coupling strength γ_R leads to moving the satellite peaks of T_A without changing their amplitudes. Moreover, when increasing ε_d, the width of the central peak becomes broader with increasing γ_R which leads to the aforementioned behavior of electrical conductance. In turn, the Seebeck coefficient is reduced compared to the symmetric case, whereas the heat conductance becomes enhanced due to the increase of the coupling strength with the TS.The behavior of G, κ and S then leads to the suppression of ZT which still achieves values much larger than 1, i. e. $ZT \approx 2$.

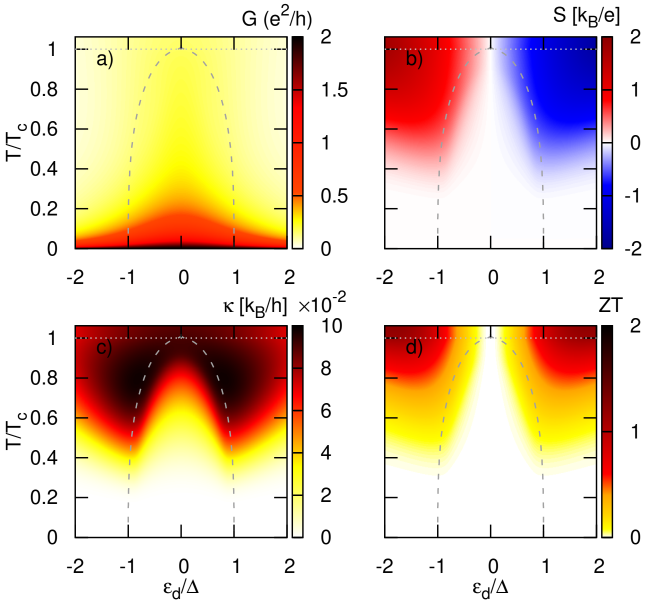

Figure 1. Thermoelectric coefficients: a) electrical conductance, b) Seebeck coefficient (thermopower), c) heat conductance, d) figure of merit, calculated as a function of the dot's energy level ε_d and temperature k_BT for $\gamma_R = 10\gamma_L = 10$ and $\Gamma = 0.1\Delta$. The dotted gray horizontal line indicates the critical temperature T_c at which the energy gap of the superconducting lead vanishes. The dashed gray line shows the temperature dependence of the superconducting energy gap $\Delta(T)$.

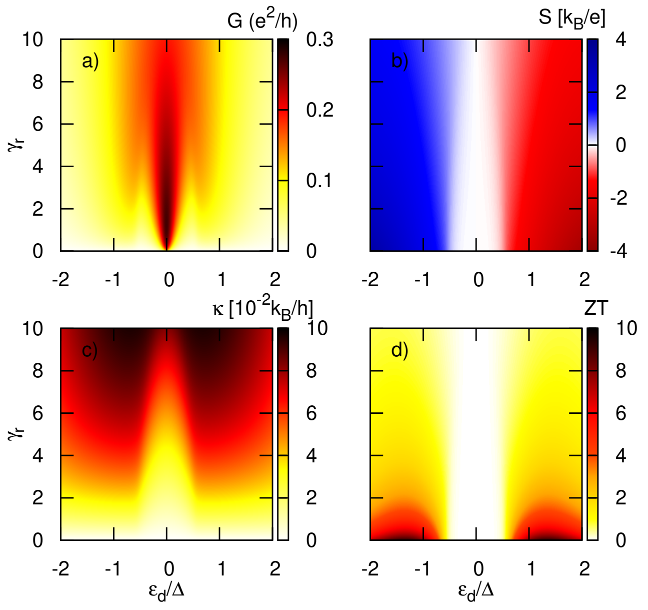

Figure 2. Thermoelectric coefficients: a) electrical conductance, b) Seebeck coefficient (thermopower), c) heat conductance, d) figure of merit, calculated as a function of the dot's energy level ε_d and asymmetry parameter γ_R for $k_BT = 0.5\Delta$, $\gamma_L = 1$ and $\Gamma = 0.1\Delta$.

To complete the study on the influence of the coupling strength to the TS, Fig. 2 shows the thermoelectric coefficients as a function of the dot energy level position and the asymmetry parameter γ_R, for a fixed temperature $k_BT/\Delta = 0.5$. These results were obtained for a relatively high temperature, which leads to a visible shrinking of the superconducting gap, as for $k_BT/\Delta = 0.5$, one obtains $\Delta(T) \approx 0.56\Delta$, i. e. al-

most halved with respect to its zero-temperature value. The electrical conductance, shown in Fig. 2(a), reveals a central resonance, associated with the ZEP and side peaks visible in T_A, and two maxima corresponding to the quasiparticle tunneling at $\sim \pm\Delta(T)$. Increasing γ_R leads to the broadening of the central peak in the electrical conductance and only slightly changing its intensity when $\gamma_R \geq \gamma_L$. This feature has already been described above. In turn, the amplitudes of the peaks in G related to quasiparticle tunneling, with maxima at $\sim \pm\Delta(T)$, grows with increasing γ_R. Additionally, for larger γ_R, the satellite peaks (resulting from the T_A term) overlap with the quasiparticle bands, further enhancing the electrical conductance. The Seebeck coefficient, presented in Fig. 2(b), shows a slow decrease as the asymmetry factor γ_R grows and becomes strongly suppressed inside the SC gap regardless of γ_R. In turn, the heat conductance monotonically increases with γ_R. Moreover, both S and κ leak deeper in the SC gap for larger asymmetry parameter γ_R. Stronger coupling to the TS electrode causes larger quasiparticle tunneling rates, and thus, greater energy transfer which explains the enhancement of κ even deep in the SC gap. The resulting figure of merit ZT, shown in Fig. 2(d), reaches its highest values for small coupling to TS electrode, i. e. for $\gamma_R \ll 1$.

Heat engine - supplementary results

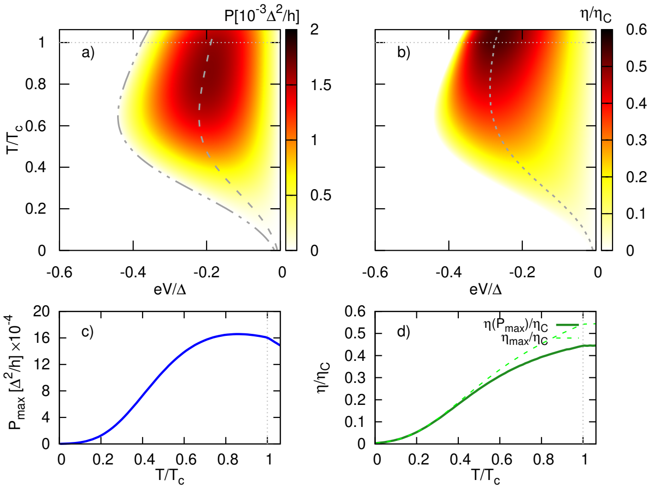

Figure 3. Bias voltage and temperature dependance of a) output power, b) the corresponding thermal efficiency normalized to Carnot's efficiency, calculated for $\varepsilon_d = 1.2\Delta$, $\Delta T = 0.2\Delta/k_B$. Dashed blue [green] line in a) [b)] indicates maximum power [maximum efficiency], whereas dash-dot gray line in a) indicates stopping (blocking) voltage. Corresponding maximum power c), efficiency at maximum power and maximum efficiency d) as a function of dot's energy level Other parameters: $\gamma_L = \gamma_R = 1$ and $\Gamma = 0.1\Delta$.

The extracted power and the corresponding efficiency can be further optimized by varying various system's parameters including ambient temperature, temperature difference set between the electrodes, and coupling strength of the dot to the leads. Both increase of temperature T and/or the temperature difference ΔT leads to enhancement of the maximum power, maximum efficiency and the efficiency at maximum power in the SC phase. Specifically, η_{max} and $\eta(P_{max})$ grow monotonically with increasing temperature up to $T = T_c$, and above T_c are almost saturated in the presented range for $T > T_c$ as shown in Fig. 3 d). In the same time, P_{max} increases achieving maximum for $T_{max} \approx 0.85T_c$, and drops with further increase of T. However, the respective drop of P_{max} for $T > T_c$ is much faster than that observed for $T_{max} < T < T_c$. This clearly indicates that greater power can be extracted in the SC phase that in the normal phase with quite similar efficiency, approximately equal to $0.45\eta_C$. Note that $\eta(P_{max})/\eta_C$ for the presented range of $T \geq T_C$ is roughly constant. For the temperature range, $k_BT < 0.25\Delta$, one notes that $\eta_{max} = \eta(P_{max})$ for $\Delta T = 0.2\Delta/k_B$. Operation of the heat engine can be also optimized by tuning the dot's coupling strength to the normal electrode keeping unchanged coupling to TS reservoir as presented in Fig. 4. When the coupling to NM lead is weak, $\gamma_L \ll 1$, the maximum output power becomes suppressed, whereas the corresponding efficiencies, η_{max} and $\eta(P_{max})$, become maximized. More precisely, for $\gamma_L \to 0$, the maximum power tends to zero, $P_{max} \to 0$, whereas $\eta_{max} \to 0.3\eta_C$ and simultaneously $\eta(P_{max})$ achieves its maximum. Almost in the whole range of γ_L, η_{max} and $\eta(P_{max})$ are the same instead of a small difference visible for $\gamma_L < 1$ which becomes more pronounced as $\gamma_L \to 0$. Slight increase of γ_L (from zero) leads to almost sudden jump of P_{max}, which achieves maximum for $\gamma_L \approx 0.8$ and then slowly decrease with further growth of γ_L. In turn, the efficiencies monotonically decrease with increasing γ_L. These results show that the best performance can be achieved for γ_L being around 0.7, whereas for $\gamma_L > 1$ the heat engine ceases to work effectively as the resulting $ZT < 1$ e. g. taking $\gamma_L \approx 2$, then $\eta(P_{max}) \approx 0.1$ and $ZT \approx 0.5$. Although the power is relatively significant for this coupling, it costs rather much work to be extracted.

Finally, Fig. 5 presents operational performance of the device when coupling strength to the TS reservoir is changed while keeping constant coupling to the normal metal electrode. Maximum power is nonmonotonic function of the parameter γ_R. It rises with increasing coupling γ_R until achieving maximum at $\gamma_R \approx 2$, and then, very slowly decreases. In turn, the corresponding efficiencies, η_{max} and $\eta(P_{max})$, slowly decreases with increasing γ_R. Similarly to the previous case, η_{max} and $\eta(P_{max})$, are the same in a broad range of γ_R and differ only slightly for $\gamma_R < 1$. Although maximum power achieves relatively large values for broad range of γ_R, the corresponding efficiencies are rather small. The efficiency for which $ZT > 1$ can be achieved for $\gamma_R < 0.5$. However, for $\gamma_R \approx 0.5$ still sufficiently high power can be extracted i. e. maximum power for $\gamma_R = 0.5$ is 0.6 of the maximal value of the P_{max}.

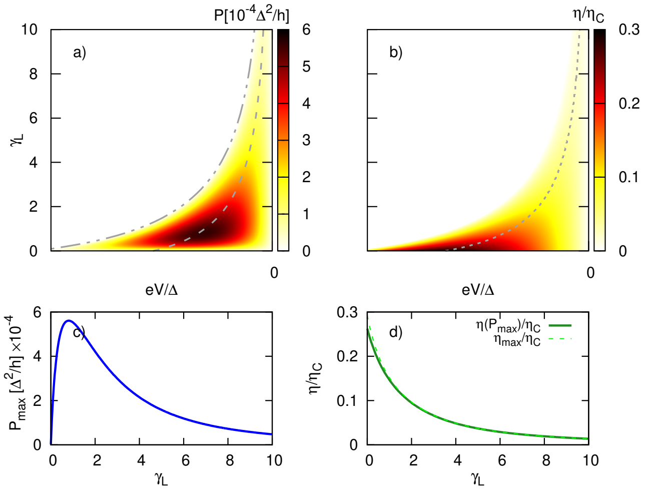

Figure 4. Power a), and the corresponding thermal efficiency normalized to Carnot's efficiency b) as a function of bias voltage and parameter γ_L, calculated for $\varepsilon_d = 1.2\Delta$, $k_B T = 0.2\Delta$ $\Delta T = 0.2\Delta/k_B$. Dashed blue [green] line in a) [b)] indicates maximum power [maximum efficiency], whereas dash-dot gray line in a) indicates stopping (blocking) voltage. Corresponding maximum power c),and efficiency at maximum power/maximum efficiency d) as a function of dot's energy level Other parameters: $\gamma_R = 1$ and $\Gamma = 0.1\Delta$.

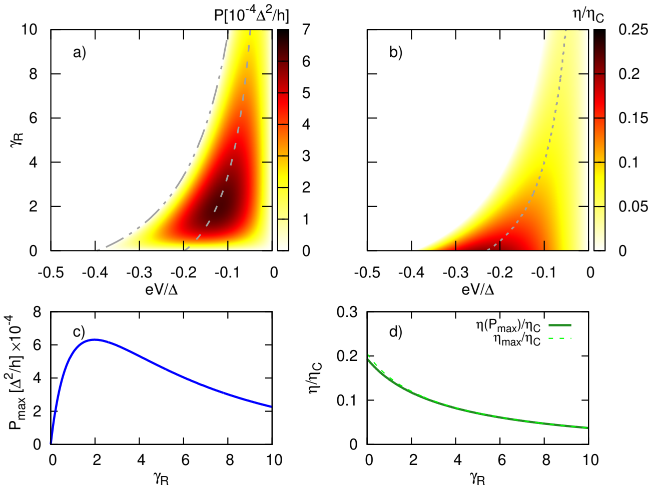

Figure 5. Power a), and the corresponding thermal efficiency normalized to Carnot's efficiency b) as a function of bias voltage and parameter γ_L, calculated for $\varepsilon_d = 1.2\Delta$, $k_B T = 0.2\Delta$ $\Delta T = 0.2\Delta/k_B$. Dashed blue [green] line in a) [b)] indicates maximum power [maximum efficiency], whereas dash-dot gray line in a) indicates stopping (blocking) voltage. Corresponding maximum power c),and efficiency at maximum power/maximum efficiency d) as a function of dot's energy level Other parameters: $\gamma_L = 1$ and $\Gamma = 0.1\Delta$.

Transmission coefficient for QD coupled to normal metal and s-wave superconductor

Here, we show transmission coefficients, T_A and T_S, calculated for hybrid junction NM-QD-SC with *s-wave* superconductor and for indicated parameters. The results can be directly compared with Fig. 2 in the main text as the same parameters were assumed. There can be noticed remarkable differences between behavior of transmission function for the two distinct systems. Particularly, for $\varepsilon_d = 0$ in SC setup single peak emerges at $\varepsilon = 0$, whereas for TS system three peak structure appear. Let us remark that single peak for SC system will split into two peak for larger coupling to SC electrode (not shown here). More interestingly, for $\varepsilon_d > 0$, single peak splits into two-peak structure for SC system. The corresponding intensities decreases. In turn, for TS setup, the central peak is immune for variation in ε_d and only side peaks move away and their amplitudes drop. Thus, the behavior of the side peaks is similar for both setups. However, only for SC system, narrow features appear near $\varepsilon = \pm\Delta$ as $\varepsilon_d \gg 0$. On the other side, the behavior of transmission above the gap looks similar for both cases besides the subtle difference in the shapes of the peaks, especially close to $\varepsilon = \Delta$, which results from different energy dependence of density of states for SC and TS.

Thermoelectric coefficients for NM-QD-TS and NM-QD-SC

In this section we show thermoelectric coefficients as a function of dot's energy level for for NM-QD-TS and for NM-QD-SC setups. Fig. 7 and Fig. 8 are cross sections extracted from Fig. 5 and Fig. 8 from the main text, respectively. In turn, Fig. 9 and Fig. 10 calculated for SC setup, corresponds to the above two plots of TS system.

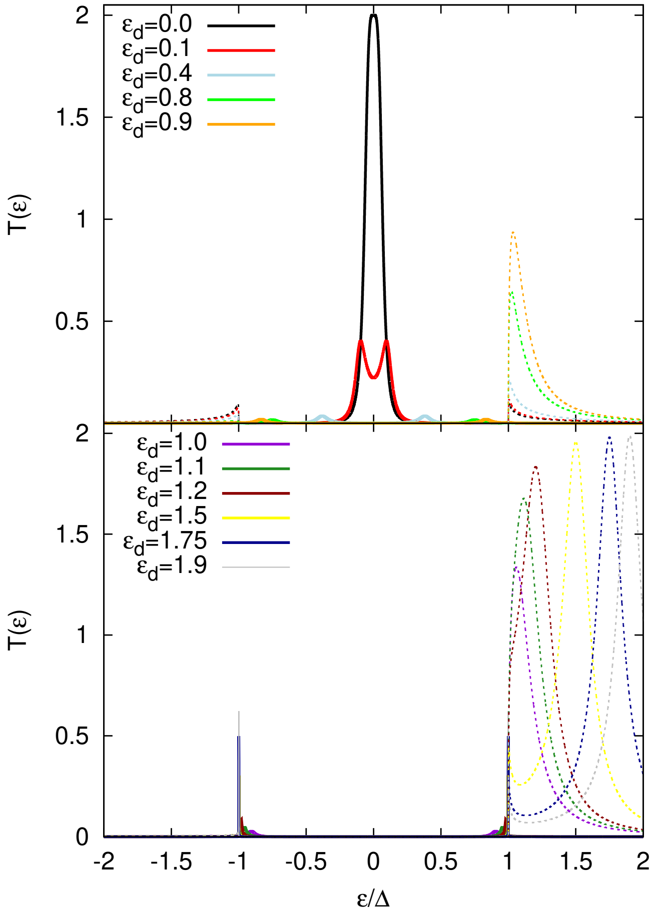

Figure 6. Transmission coefficients as a function of energy calculated for the indicated values of the dot's energy level and for symmetric coupling to the leads, $\gamma_L = \gamma_R = 1$ for QD coupled to normal metal and s-*wave* superconductor. Solid lines correspond to $T_A(\varepsilon)$, whereas dotted lines are associated with $T_S(\varepsilon)$. Other parameters: $\Gamma = 0.1\Delta$, $T = 0$.

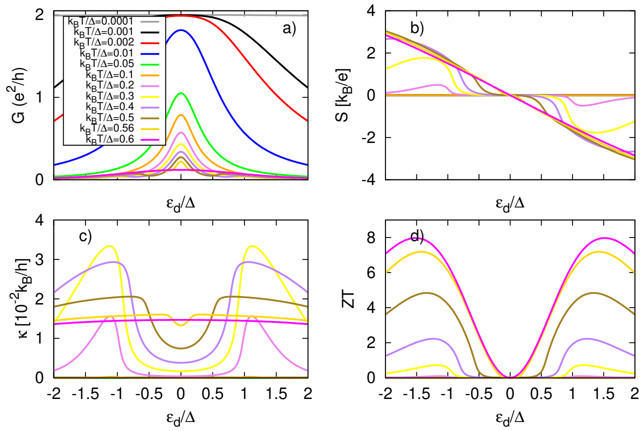

Figure 7. Thermoelectric coefficients for NM-QD-TS: a) electrical conductance, b) Seebeck coefficient (thermopower), c) heat conductance, d) figure of merit, calculated as a function of the dot's energy level ε_d for indicated values of temperature k_BT, and for $\gamma_R = \gamma_L = 1$ and $\Gamma = 0.1\Delta$. Note that for small temperatures S, κ and ZT become obscured in the plots.

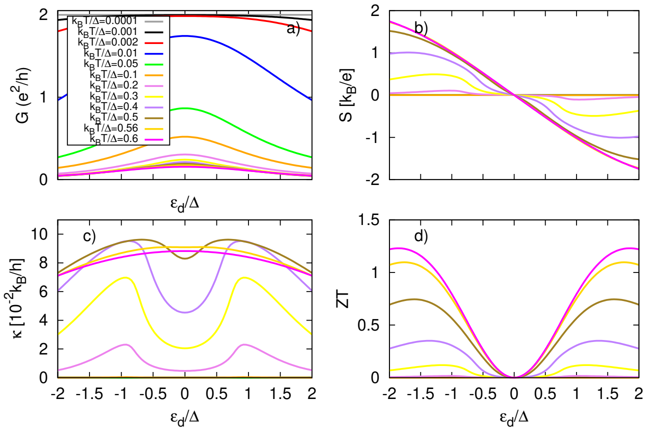

Figure 8. Thermoelectric coefficients for NM-QD-TS: a) electrical conductance, b) Seebeck coefficient (thermopower), c) heat conductance, d) figure of merit, calculated as a function of the dot's energy level ε_d for indicated values of temperature k_BT, and for $\gamma_R = 10$, $\gamma_L = 1$ and $\Gamma = 0.1\Delta$.

Nonequilibrium regime – Differential conductance

Here we present our results for the differential conductance as a function of bias voltage eV and dot's energy level ε_d for low and high temperature regimes and for symmetric and strongly asymmetric coupling situations. The differential conductance, G, is defined as the derivative of the electrical current with respect to the applied bias voltage under condition of zero temperature difference;

$$G = \left.\frac{\mathrm{d}J_e}{\mathrm{d}V}\right|_{\Delta T=0}. \tag{1}$$

Let us first consider the zero-temperature limit and then we will describe the influence of finite temperature. When the dot's energy level is situated in the SC gap, $|\varepsilon_d| < \Delta$, the differential conductance reveals three peaks, in general. These maxima are associated with the sub-gap tunneling processes. The central zero-bias peak (ZBP) reaches the maximal allowed intensity equal to two quanta of conductance for the whole range of dot's energy level. Indeed, it is also present for $|\varepsilon_d| > \Delta$, although in Fig. 11(a) due to the finite resolution, the results for larger $|\varepsilon_d|$ become obscured. Zero-bias anomaly is a consequence of Majorana bound states hold by the TS. In turn, the intensity of the satellite peaks reaches its maximal value only for $\varepsilon_d = 0$ and rapidly decreases as the dot's level position moves away from resonance. These sub-gap peaks are due to the dot's proximity to the TS electrode and result from virtual tunneling processes resembling Andreev bound

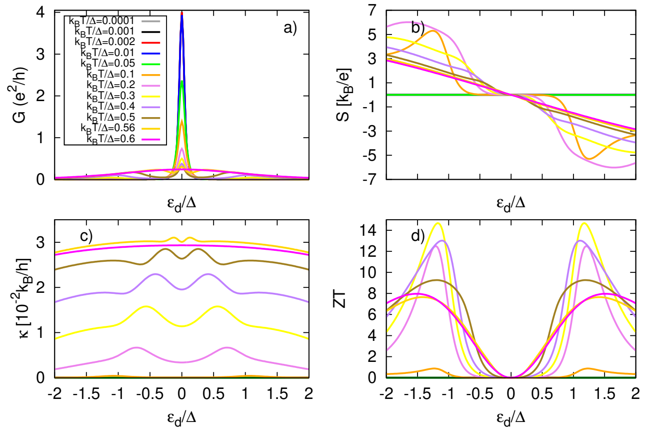

Figure 9. Thermoelectric coefficients for NM-QD-SC: a) electrical conductance, b) Seebeck coefficient (thermopower), c) heat conductance, d) figure of merit, calculated as a function of the dot's energy level ε_d for indicated values of temperature $k_B T$, and for $\gamma_R = \gamma_L = 1$ and $\Gamma = 0.1\Delta$.

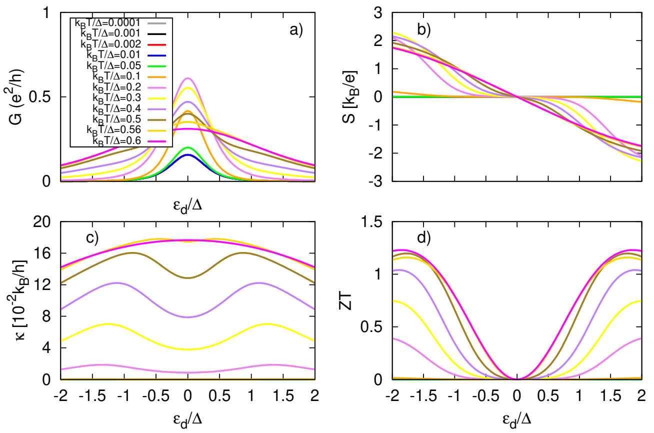

Figure 10. Thermoelectric coefficients for NM-QD-SC: a) electrical conductance, b) Seebeck coefficient (thermopower), c) heat conductance, d) figure of merit, calculated as a function of the dot's energy level ε_d for indicated values of temperature $k_B T$, and for $\gamma_R = 10$, $\gamma_L = 1$ and $\Gamma = 0.1\Delta$.

states in NM-QD-SC system. In turn, coupling to the normal metal reservoir leads to broadening of these resonances. Notice that ZBP broadening diminishes as the dot's level moves away from zero energy, whereas its position is unchanged due to particle-hole symmetry. Moreover, as $\varepsilon_d \to \infty$ the ZBP becomes totally localized. The differential conductance related to quasiparticle tunneling becomes the most pronounced in the regions determined by $|\varepsilon_d| > \Delta$ and $|eV| > \Delta$.

Stronger coupling to the TS reservoir changes the above picture both quantitatively and qualitatively. One can notice in Fig. 11(d) that the three-peak structure in the sub-gap region becomes well resolved as side-peaks move away from the central one. Simultaneously, the width of the central peak slightly grows for $\varepsilon_d \neq 0$ in comparison to the symmetric coupling situation. Moreover, side-peaks' intensities don't decrease so rapidly as $|\varepsilon_d|$ increases. In turn, features outside the gap become more pronounced.

Finite temperature has a profound impact on the differential conductance, especially in the sub-gap region. Firstly, the sub-gap peaks structure merges into a single maximum centered at zero bias voltage. Secondly, the intensities of this peak as well as the features outside the gap diminish with increasing temperature as a result of the Fermi-Dirac temperature dependence. The smoothing of the Fermi-Dirac function leads to a redistribution of electrons (and holes) in a broader range of energies, and thus, to a smaller population of carriers available for a given bias voltage. Surprisingly, the zero-bias peak in differential conductance is rapidly suppressed when moving the dot's level position away from zero energy. The temperature dependence of the Fermi-Dirac distribution together with the localization of the MBS explain this behavior.

On the other hand, when the QD is much strongly coupled to the TS reservoir, an increase of the temperature leads to greater broadening of the differential conductance features compared to the symmetric coupling case. Moreover, the intensity of the sub-gap peak becomes reduced, whereas the maxima outside the gap are greater than the corresponding features in the equal coupling situation. The latter feature can be explained as follows: increasing the coupling to the TS electrode directly leads to an enhancement of the tunneling amplitude of quasiparticles. In turn, sub-gap tunneling processes occur via virtual tunneling events due to proximity with the normal metal electrode and strongly depend on the coupling strength to this reservoir, whereas the coupling to the TS lead can only indirectly influence the ZBP. For sufficiently large coupling to the TS electrode (compared to γ_L), the side-peaks in the transmission become well resolved from the central peak, and thus, only add a vanishingly small contribution to the zero-bias conductance, whereas for $\gamma_L = \gamma_R$ they contribute more to the ZBP as they are close to the MBS.

Power factor and ZT – comparison

Another thermoelectric quantity, power factor $P = GS^2$, also determines the potential utilization of the device. It indicates the power that can be extracted, while ZT determines the efficiency of power extraction. Thus, one need to optimize both quantities. It turns out that P is the largest just below T_c, and decreasing with further increase of T, as shown in Fig. 12. Notice that P suddenly and almost linearly drops above T_c, whereas ZT increases at a slower rate. Moreover, the increase of ZT is notably weaker than linear. Notice also that a linear increase of ZT with temperature follows from its definition, $ZT = (GS^2/\kappa)T$.

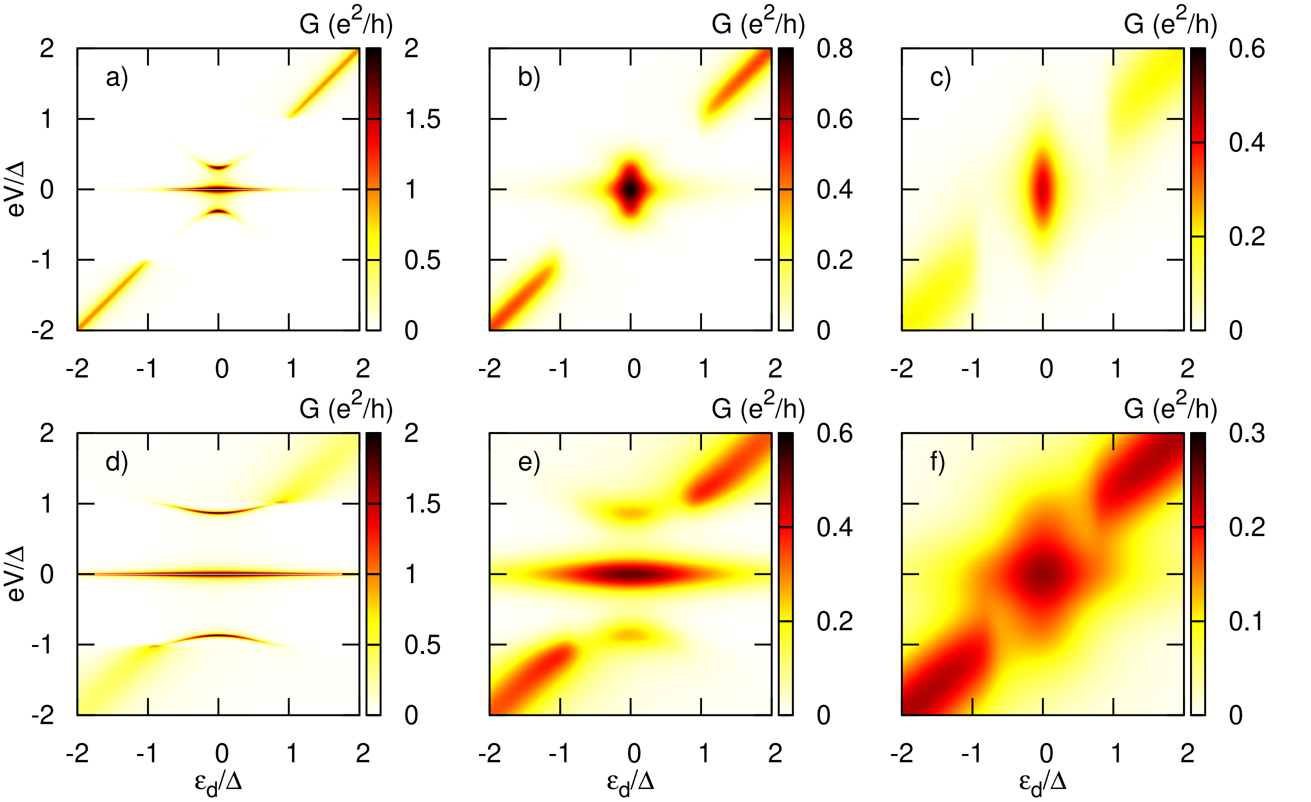

Figure 11. Differential conductance as a function of dot's energy level and bias voltage calculated for a) $k_BT = 0$ and $\gamma_R = 1$, b) $k_BT = 0.1\Delta$ and $\gamma_R = 1$, c) $k_BT = 0.3\Delta$ and $\gamma_R = 1$, d) $k_BT = 0$ and $\gamma_R = 10$, e) $k_BT = 0.1\Delta$ and $\gamma_R = 10$, f) $k_BT = 0.3\Delta$ and $\gamma_R = 10$. The other parameters are: $\gamma_L = 1$, $\Gamma = 0.1\Delta$.

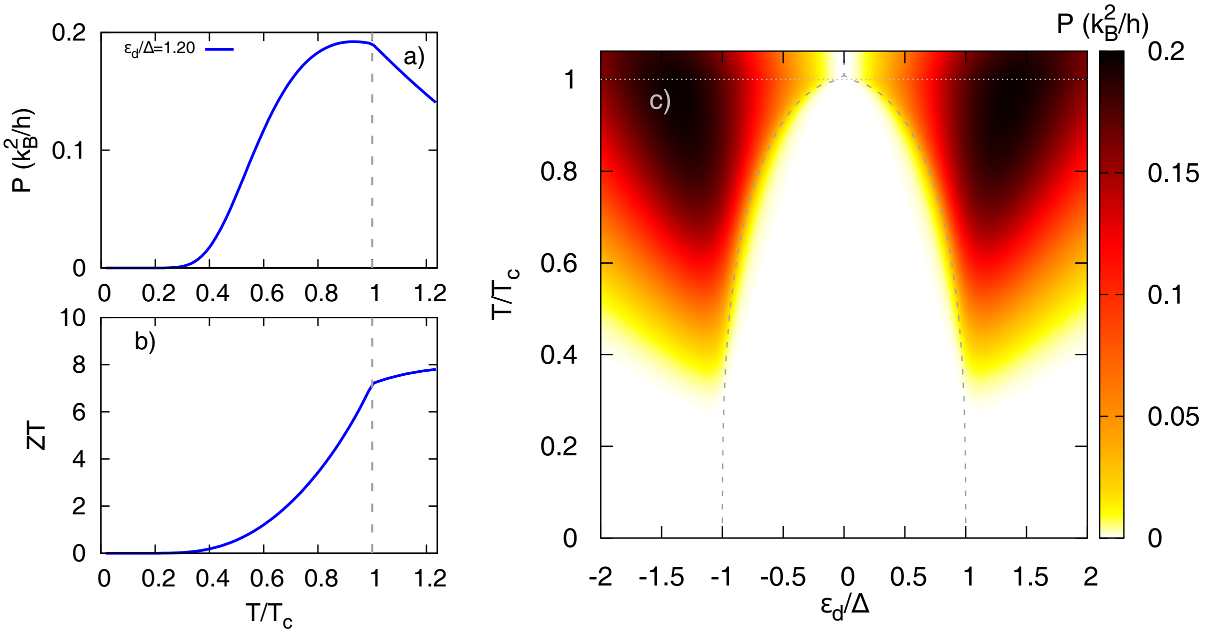

Figure 12. a) Power factor P, b) ZT as a function of temperature calculated for $\varepsilon_d = 1.2\Delta$, c) power factor as a function of dot's energy level ε_d and temperature T. Dashed horizontal, in a) and b), and vertical in c) line indicates critical temperature T_c.

Explanation of transmission behavior

Quasiparticle (QP) tunneling in the negative energy region becomes increasingly suppressed as ε_d shifts from zero toward positive values ($\varepsilon_d > 0$). The energy level of the quantum dot (QD), which is coupled to the normal metal (NM) electrode, acquires finite broadening.

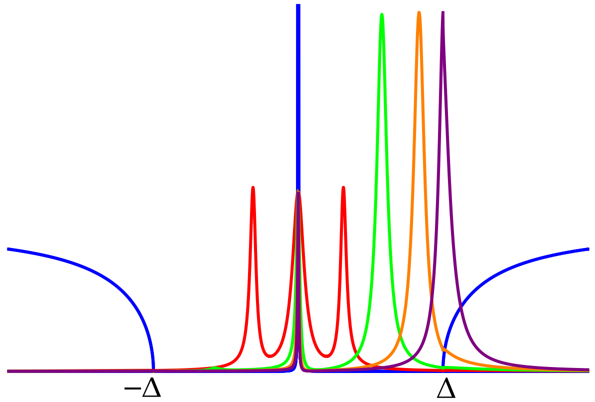

Figure 13. Density of states (DOS) of the TS (blue line) and full DOS of QD coupled to the NM and TS electrodes calculated for $\gamma_L = \gamma_R = 1$, and for $\varepsilon_d = 0$ (red), $\varepsilon_d = 0.5\Delta$ (green), $\varepsilon_d = 0.8\Delta$ (orange), $\varepsilon_d = \Delta$ (purple).

Here, we provide an explanation of transmission behavior with change in dot's energy level based on the full density of states (DOS), shown in Fig. 13. The DOS for $\varepsilon_d = 0$ consists of three peaks (in red) of equal amplitude, symmetrically located around zero energy. While the tails of this peak structure are vanishingly small, they still contribute equally to tunneling for energies above ($\varepsilon > \Delta$) and below ($\varepsilon < \Delta$) the superconducting (SC) gap.

Shifting the dot's energy level away from zero significantly alters the DOS. The further ε_d moves from zero (considering positive ε_d here), the narrower the central peak becomes, which explains the shrinking of the central peak in transmission. At the same time, the side peaks no longer have equal amplitudes: the amplitude of the right peak in the DOS gradually increases until it saturates, while the amplitude of the left peak diminishes progressively (until totally suppressed). For $\varepsilon_d = 0.5\Delta$, only a small feature related to the left peak remains visible.

This shift also explains the suppression of subgap side peaks in the transmission, which result from Andreev processes. Both peaks in the DOS are necessary for electron transport via Andreev reflections, meaning that the tunneling is primarily governed by the smaller peak. Meanwhile, as ε_d increases, the tail of the right peak increasingly overlaps with the TS density of states for $\varepsilon > \Delta$, leading to enhanced QP tunneling. Conversely, the left peak becomes increasingly suppressed as ε_d grows, reducing the overlap of its tail with the TS density of states for $\varepsilon < \Delta$, and thus, suppressing the corresponding transmission. As a result, QP tunneling increases for $\varepsilon \geq \Delta$ and decreases for $\varepsilon \leq -\Delta$ as ε_d increases. Finally, when $\varepsilon_d > \Delta$, the entire dot resonance overlaps with QP states at energies $\varepsilon \geq \Delta$, leading to a clear peak in QP tunneling.
